# Follistatin-Like-1 (FSTL1) Is a Fibroblast-Derived Growth Factor That Contributes to Progression of Chronic Kidney Disease

**DOI:** 10.3390/ijms22179513

**Published:** 2021-09-01

**Authors:** Nicholas A. Maksimowski, Xuewen Song, Eun Hui Bae, Heather Reich, Rohan John, York Pei, James W. Scholey, Nephrotic Syndrome Study Network (NEPTUNE)

**Affiliations:** 1 Institute of Medical Science, University of Toronto, Toronto, ON M5S 1A8, Canada; york.pei@uhn.ca (Y.P.); james.scholey@utoronto.ca (J.W.S.); 2 Division of Nephrology, University Health Network, Toronto, ON M5G 2C4, Canada; xuewen.song@utoronto.ca (X.S.); heather.reich@uhn.ca (H.R.); 3 Departments of Internal Medicine, Chonnam National University Medical School, Gwangju 61469, Korea; baedak7@gmail.com; 4 Toronto General Hospital Research Institute, University Health Network, Toronto, ON M5G 2C4, Canada; rohan.john@uhn.ca; 5 Department of Laboratory Medicine and Pathobiology, University of Toronto, Toronto, ON M5G 2C4, Canada; 6 Department of Pathology, University Health Network, Toronto, ON M5G 2C4, Canada; 7 Department of Physiology, University of Toronto, Toronto, ON M5G 2C4, Canada

**Keywords:** kidney, FSTL1, fibrosis, inflammation, cytokines, apoptosis, nephrotic syndrome

## Abstract

Our understanding of the mechanisms responsible for the progression of chronic kidney disease (CKD) is incomplete. Microarray analysis of kidneys at 4 and 7 weeks of age in *Col4a3^-/-^* mice, a model of progressive nephropathy characterized by proteinuria, interstitial fibrosis, and inflammation, revealed that Follistatin-like-1 (*Fstl1*) was one of only four genes significantly overexpressed at 4 weeks of age. mRNA levels for the *Fstl1* receptors, *Tlr4* and *Dip2a*, increased in both *Col4a^-/-^* mice and mice subjected to unilateral ureteral obstruction (UUO). RNAscope^®^ (Advanced Cell Diagnostics, Newark CA, USA) localized *Fstl1* to interstitial cells, and in silico analysis of single cell transcriptomic data from human kidneys showed *Fstl1* confined to interstitial fibroblasts/myofibroblasts. In vitro, FSTL1 activated AP1 and NFκB, increased collagen I (COL1A1) and interleukin-6 (IL6) expression, and induced apoptosis in cultured kidney cells. *FSTL1* expression in the NEPTUNE cohort of humans with focal segmental glomerulosclerosis (FSGS), membranous nephropathy (MN), and IgA nephropathy (IgAN) was positively associated with age, eGFR, and proteinuria by multiple linear regression, as well as with interstitial fibrosis and tubular atrophy. Clinical disease progression, defined as dialysis or a 40 percent reduction in eGFR, was greater in patients with high baseline *FSTL1* mRNA levels. *FSTL1* is a fibroblast-derived cytokine linked to the progression of experimental and clinical CKD.

## 1. Introduction

The prevalence and progression of chronic kidney disease (CKD) remains a global clinical challenge, and the development of end stage kidney disease is a costly outcome requiring therapies like dialysis and kidney transplantation. This underscores the important need to develop new and effective treatments to lessen the burden of CKD, but we require a better understanding of the pathogenesis of CKD progression in order to identify new targets for therapy [1].

The decline in glomerular filtration in CKD, including diseases that affect the kidney glomerulus, is associated with pathology in the kidney tubulointerstitium. These changes include interstitial inflammation and fibrosis, loss of the peritubular capillary network and loss of tubular epithelial cell number and volume, recognized as tubular atrophy [1,2]. Indeed, tubulointerstitial fibrosis strongly correlates with GFR decline in a number of kidney diseases including glomerulopathies [3]. Blockade of the renin angiotensin system remains the first line approach to limiting progression of CKD and reducing end stage kidney disease (ESKD), especially in the setting of proteinuria, while new data suggest that the use of sodium glucose co-transport-2 (SGLT2) inhibitors may affect progression broadly, and this is an active area of ongoing investigation [4]. There remains an unmet need for new and targeted therapies to slow the progression of CKD towards ESKD.

In order to identify new treatment targets for CKD, we studied *Col4a3*^-/-^ mice with homozygous deletion of the gene that encodes the a3 chain of collagen IV. Mutations in genes encoding the a3.a4.a5 collagen IV network led to glomerular basement membrane (GBM) structural abnormalities that occur at the time of the normal molecular switch from the a1.a2.a1 collagen IV to the mature collagen IV. This change in basement membrane proteins leads to depletion of podocytes, progressive glomerular sclerosis, tubulointerstitial inflammation and fibrosis, and the development of kidney failure [5,6]. Although developed as a model of Alport Syndrome, the disease phenotype re-capitulates classic features of proteinuric CKD in humans. In this regard, recent clinical studies have implicated collagen IVa3 in the pathogenesis of some forms of focal segmental glomerulosclerosis and in the pathogenesis of diabetic nephropathy [5,7].

We performed unbiased global gene-expression profiling in kidneys of *Col4a3*^-/-^ and wild-type (WT) mice at 4 and 7 weeks of age. Surprisingly, we identified only four differentially expressed genes in 4 week old mice. Follistatin-like-1 (*Fstl1*) was one of the genes, and the early increase in expression persisted through to 7 weeks of age. Studies have implicated FSTL1 in lung development and fibrosis, as well as cardiac injury, but information about its role in the pathogenesis of experimental CKD is incomplete [8,9,10,11]. Moreover, there are no studies of FSTL1 in humans with CKD associated with proteinuria.

In the current report, we sought to address this gap. We studied the expression and localization of FSTL1 in *Col4a3*^-/-^ mice, and related expression to genes implicated in kidney fibrosis, inflammation, and apoptosis. We also studied the effect of FSTL1 on cultured human kidney cells, and then extended the work to mice with unilateral ureteral obstruction. Finally, we utilized human transcriptomic data as well as functional and structural data from the NEPTUNE Consortium to determine if *FSTL1* expression relates to kidney function, interstitial fibrosis, and progression of CKD in humans.

## 2. Results

### 2.1. Breeding Strategy for Col4a3^-/-^ Mice and Experimental Design

**Figure 1:**
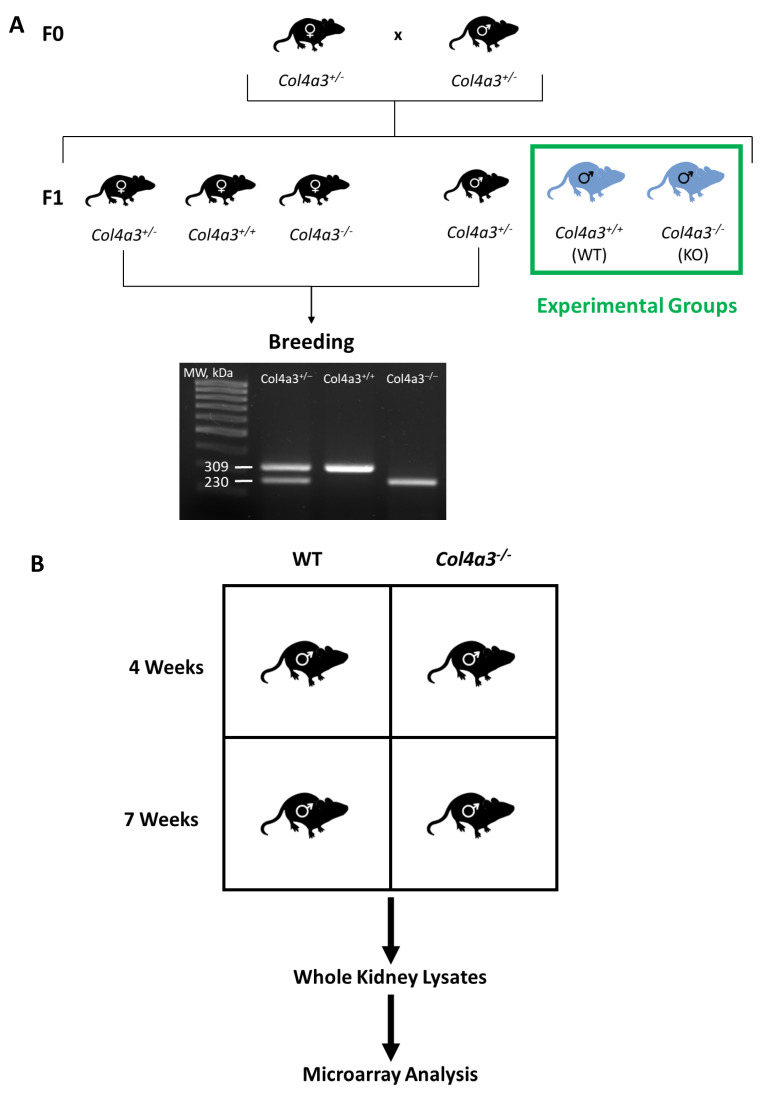
Schematic diagram summarizing experimental workflow. (**A**) breeding strategy for generating experimental groups for analysis. (**B**) Whole kidney samples from 4 and 7 week old wild type (WT) and *Col4a3*^-/-^ (KO) mice were subjected to microarray expression profiling.

We generated *Col4a3*^-/-^ (KO) and *Col4a3*^+/+^ (WT) male mice, and studied gene expression in whole kidneys from 4 and 7 week old mice (Figure 1A). Body weights and kidney weights were similar in the two groups at 4 and 7 weeks of age. KO mice exhibited a 3-fold increase in the urinary albumin excretion rate (UalbV) at 4 weeks of age (*p* < 0.05). The UalbV continued to increase in the KO mice, reaching a 7-fold increase at 7 weeks of age (Table 1).

**Figure 2:**
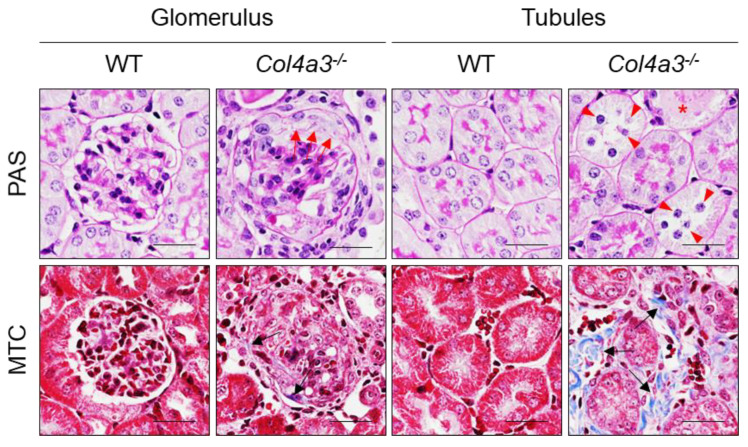
Comparisons of the Kidney Tissue Morphology of Kidney of WT and *Col4a3*^-/-^ mice. Images from glomerulus (left) and tubulointerstitium (right) are presented. Periodic Acid Schiff staining (PAS); Masson’s trichrome staining (MTC). In *Col4a3*^-/-^ mice, crescents are packing the Bowman’s space (red arrows). Detachment of tubular epithelial cells from basement membrane (red arrowheads), tubular cast (red asterisks), and collagen deposits (black arrows) are also indicated. Scale bars, 50 μm.

Figure 2 shows light micrographic images of kidneys from WT mice and *Col4a3^-/-^* mice (at 7 weeks of age). Panel A shows PAS-stained sections of glomeruli and tubules from WT mice and *Col4a3^-/-^* mice and Masson Trichome (MTC) stains to show interstitial fibrosis (green in lower right panel).

**Figure 3:**
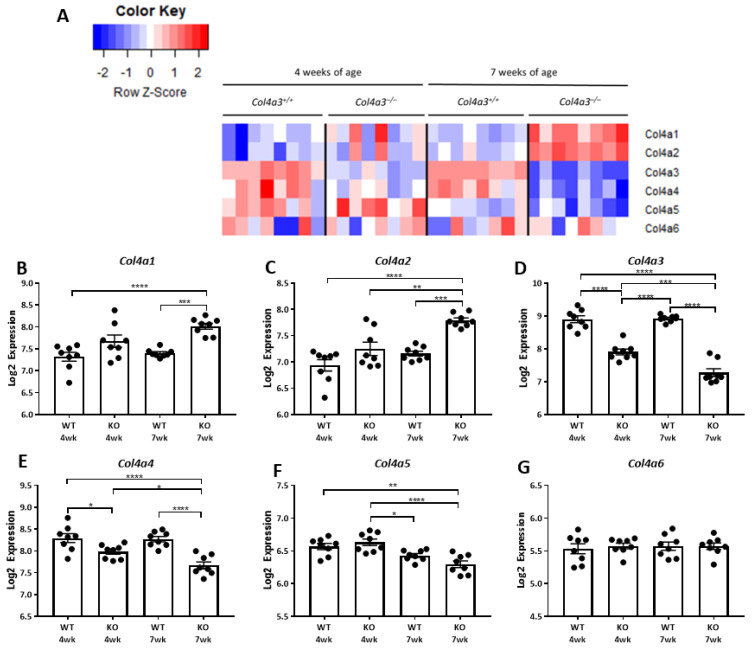
Microarray expression of collagen 4 alpha 1-6. (**A**) Heatmap with unsupervised hierarchical cluster analysis of col4 genes in kidneys of 4 and 7 week old *Col4a3*^-/-^ and wild-type mice (*n* = 8 per group). Each column reflects a kidney sample, and each row represents an individual gene. Red and blue color intensities correlate with the scaled up-regulation and downregulation of the gene, respectively. (**B**–**G**) Graphical representation of microarray expression from panel A. Values are mean ± SEM. *p* values were determined by 1-way ANOVA. * *p* value < 0.05. ** *p* value < 0.01. *** *p* value < 0.001. **** *p* value < 0.0001.

The molecular switch from the immature collagen IV network (a1a2a1) to the mature collagen IV network (a3a4a5) is evident in the heat map of collagen gene expression at 4 and 7 weeks of age in the *Col4a3*^-/-^ (KO) and *Col4a3*^+/+^ (WT) mice (Figure 3A). At 7 weeks of age *Col4a3* and *Col4a4* were more highly expressed than *Col4a1* and *Col4a2* in the *Col4a3*^+/+^ mice, but the persistence of Col4a1 and Col4a2 expression is evident in the *Col4a3*^-/-^ mice while levels of *Col4a3*, *Col4a4*, *Col4a5* and *Col4a2* are down-regulated (Figure 3B–G). The decrease in mRNA levels for *Col4a3* is expected but it is interesting to note that deletion of the gene for *Col4a3* leads to reduced expression of *Col4a4* and *Col4a5* relative to the *Col4a3*^+/+^ mice (Figure 3E,F).

**Figure 4:**
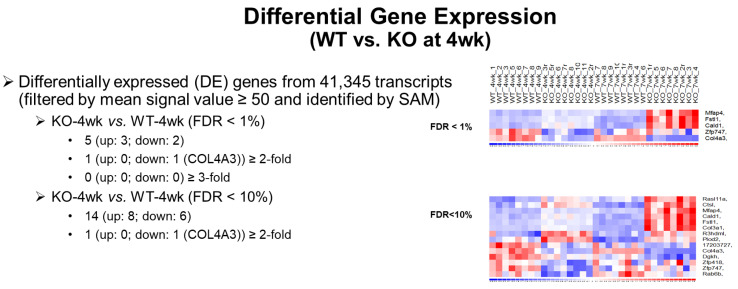
Differential Gene Expression in WT vs. KO mice at 4 Weeks of Age. Explanation of the statistical parameters used to identify the 5 differently expressed genes at 4 weeks of age in WT vs. KO mice.

**Figure 5:**
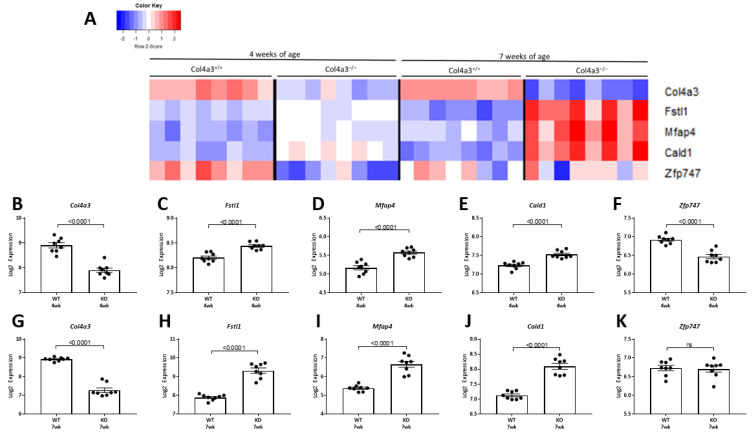
Microarray expression of genes differentially expressed at 4 and 7 weeks of age. (**A**) Heatmap with unsupervised hierarchical cluster analysis of the five genes that were differentially expressed at 4 weeks of age in the kidneys of *Col4a3*^-/-^ mice. The analysis shows the gene expression in kidneys of 4 and 7 week old *Col4a3*^-/-^ and wild-type mice (*n* = 8 per group). (**B**–**F**) Graphical representation of mRNA levels in 4 week old wild type mice versus 4 week old *Col4a3*^-/-^ mice. (**G**–**K**) Graphical representation of microarray expression in 7 week old wild type versus 7 week old *Col4a3*^-/-^ mice. Values are mean ± SEM, and significance was defined as a *p* value of < 0.05 by Student’s *t* tests.

There were five genes differentially expressed in *Col4a3*^-/-^ mice compared to *Col4a3*^+/+^ mice at 4 weeks of age. These five genes were identified using Significance Analysis of Microarrays (SAM) and a FDR of <1% (Figure 4). Expression levels at both 4 weeks (Figure 5A–F) and 7 weeks (Figure 5G–K) of age are depicted in Figure 5. As expected, *Col4a3* mRNA levels were lower in *Col4a3*^-/-^ mice compared to *Col4a3*^+/+^ mice (Figure 5A) while *Zfp747*, a zinc finger transcription factor, was also decreased in the kidneys of 4 week old *Col4a3^-/-^* mice (Figure 5F). The expression of Follistatin-like 1 (*Fstl1*), Microfibril associated protein 4 (*Mfap4*), and Caldesmon 1 (*Cald1*) were all increased in *Col4a3*^-/-^ mice compared to *Col4a3*^+/+^ mice at 4 weeks of age (Figure 5C–E) and the differential expression was also present at 7 weeks of age (Figure 5H–J). FSTL1 is an extracellular growth factor. MFAP4 is an extracellular protein implicated in cell-matrix interactions and it binds to collagen. CALD1 is an intracellular protein that binds actin and may regulate cell contraction. It is tempting to speculate that these later two up-regulated genes may serve to help stabilize the immature collagen network that persists in the *Col4a3*^-/-^ mice and facilitate the structural integrity of the glomerular podocyte. As a start, we focused on *Fstl1*.

**Figure 6:**
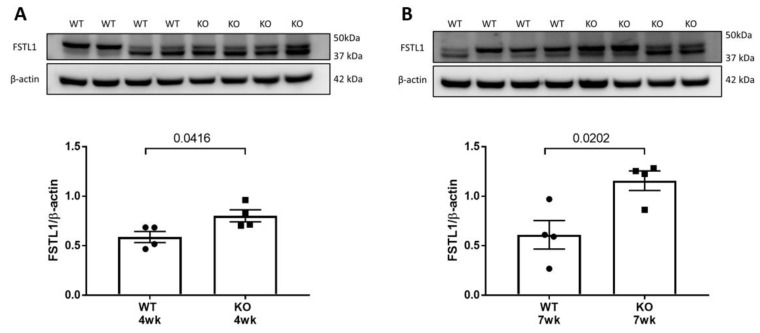
FSTL1 Protein Expression in WT and KO mouse kidney. (**A**) Representative immunoblot and quantification of FSTL1 in 4 week WT and KO mouse kidney. (**B**) Representative immunoblot and quantification of FSTL1 in 7 week WT and KO mouse kidney. Values are the mean ± SEM (black bars). *p* values were determined by Student’s *t* tests, and significance was defined as a *p* value < 0.05.

We performed Western blot analyses of the kidneys of 4 and 7 week old WT and *Col4a3* KO mice (Figure 6). There was a significant increase in the protein expression of FSTL1 at 4 and 7 weeks of age. The magnitude of change was less in the mice at 4 weeks of age, as expected from our mRNA analyses. FSTL1 is a secreted glycoprotein protein. As such, the glycosylation state plays a role in its molecular weight [12]. Therefore, both bands were used to quantify FSTL1 expression. The findings are concordant with our mRNA analyses.

### 2.2. Localization of Fstl1 in the Kidney

**Figure 7:**
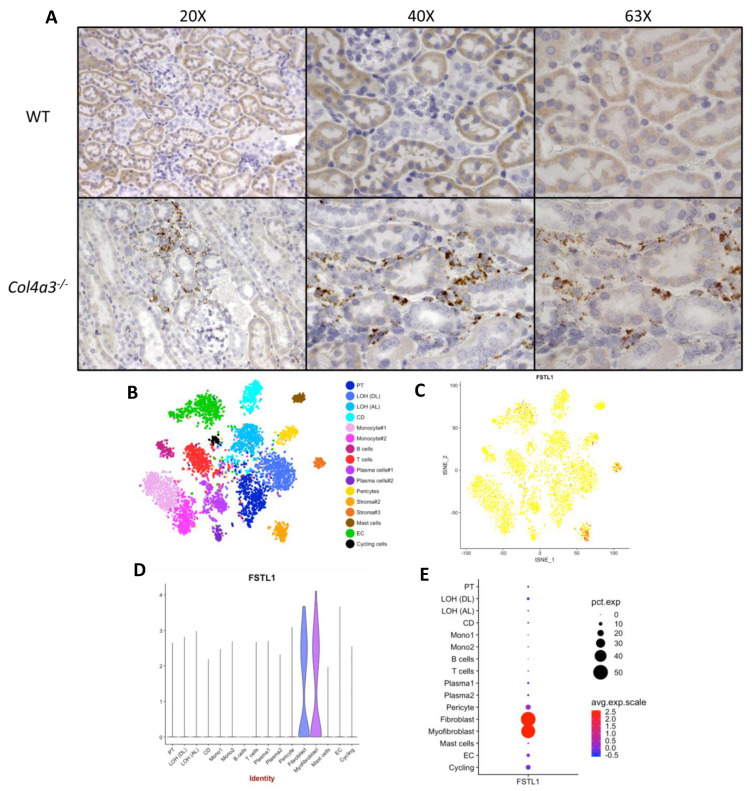
*Fstl1* expression localization in the kidney. (**A**) Light microscopic images at three magnifications of RNAscope^®^ *Fstl1* localization in wild type mice (upper three panels) and *Col4a3^-/-^* mice (lower three panels). (**B**–**E**) Cell Clustering and *Fstl1* expression from the Kidney Interactive Transcriptomics (KIT), human rejecting kidney allograft biopsy cells (http://humphreyslab.com/SingleCell, accessed on 15 March 2021).

To identify the cellular origin of FSTL1 expression we first used RNAscope^®^ to localize *Fstl1* in 7 week old *Col4a3*^-/-^ and *Col4a3*^+/+^ mice. Figure 7A shows representative light micrographs. Very little expression was identified in the *Col4a3*^+/+^ mice but in accord with the microarray analysis, there was a marked increase in *Fstl1* expression in the kidneys of the 7 week old *Col4a3*^-/-^ mice. The cells expressing *Fstl1* were present in the kidney cortex and uniformly in the interstitial space, either in capillary endothelial cells, pericytes, or resident interstitial fibroblasts. We then studied publicly available single single-cell RNA sequencing (scRNA-seq) data from human kidneys with transplant nephropathy. Figure 7B–E shows this analysis. *Fstl1* expression localized to fibroblasts and myofibroblasts in the kidney, with very little expression in pericytes or endothelial cells in this human dataset.

### 2.3. Expression of Cognate Receptors for FSTL1 in the Kidney

**Figure 8:**
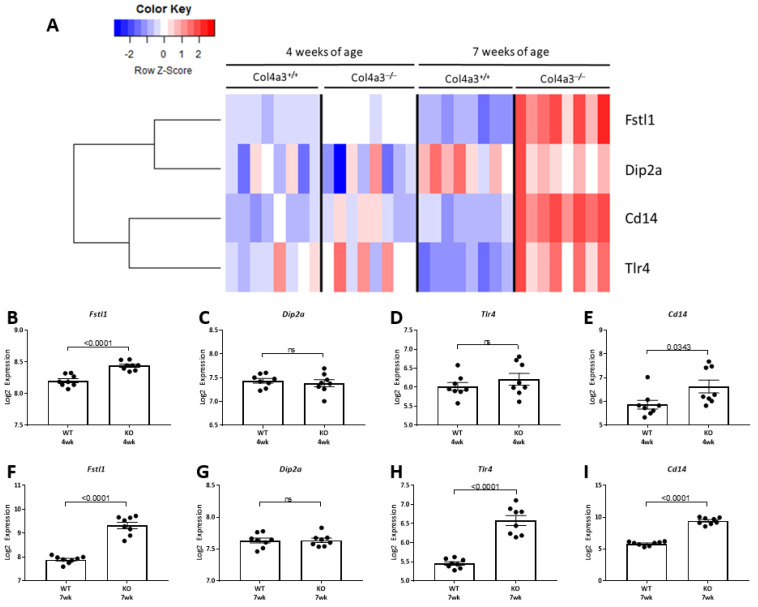
*Fstl1* and cognate receptor expression. (**A**) Heatmap with unsupervised hierarchical cluster analysis of *Fstl1*, *Dip2a*, *Cd14*, and *Tlr4* genes in kidneys of 4 and 7 week old *Col4a3*^-/-^ and wild-type mice (*n* = 8 per group). (**B**–**E**) Graphical representation of microarray expression in 4 week old wild type versus 4 week old *Col4a3*^-/-^ mice. (**F**–**I**) Graphical representation of microarray expression in 7 week old wild type versus 7 week old *Col4a3*^-/-^ mice. Values are mean ± SEM, and significance was defined as a *p* value of < 0.05 by Student’s *t* tests.

FSTL1 signal transduction involves three receptor proteins: TLR4, CD14 and DIP2A. This led us to examine expression levels of these putative cognate receptors. As illustrated in the heat map and analyses in Figure 8A, we identified transcript levels for all three proteins. Expression levels of *Tlr4* and *Cd14* are significantly higher in the kidneys of 4 and 7 week old *Col4a3*^-/-^ mice compared to *Col4a3*^+/+^ mice (Figure 8D,E,H,I). There was no significant increase in *Dip2a* expression at either time point (Figure 8C,G).

**Figure 9:**
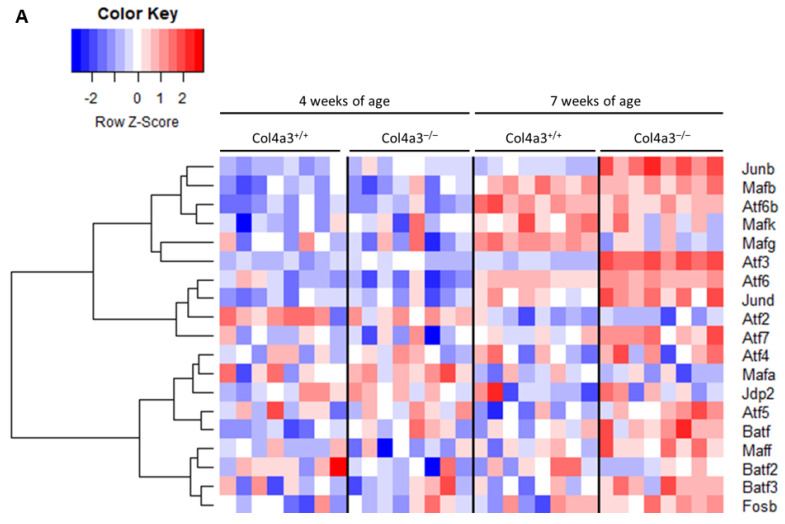
AP1 Heatmap. (**A**) Heatmap with unsupervised hierarchical cluster analysis of AP1 genes in kidneys of 4 and 7 week old *Col4a3*^-/-^ and wild-type mice (*n* = 8 per group).

AP1 is a transcription factor that is a dimer of a family of proteins and the most classical dimer is composed of the proteins FOS and JUN, characterized as early response genes. We utilized a list of genes for proteins of the AP1 family [13] and then performed an unsupervised hierarchical cluster analysis of AP1 gene expression in the kidneys of our mice. Figure 9 depicts the expression levels of these genes. There is a generalized but not universal upregulation of the expression of these AP1 genes at 7 weeks of age in the *Col4a3* KO mice.

### 2.4. p42/44 MAPK and Activator Protein 1 (AP1) Signaling in Response to rhFSTL1

**Figure 10:**
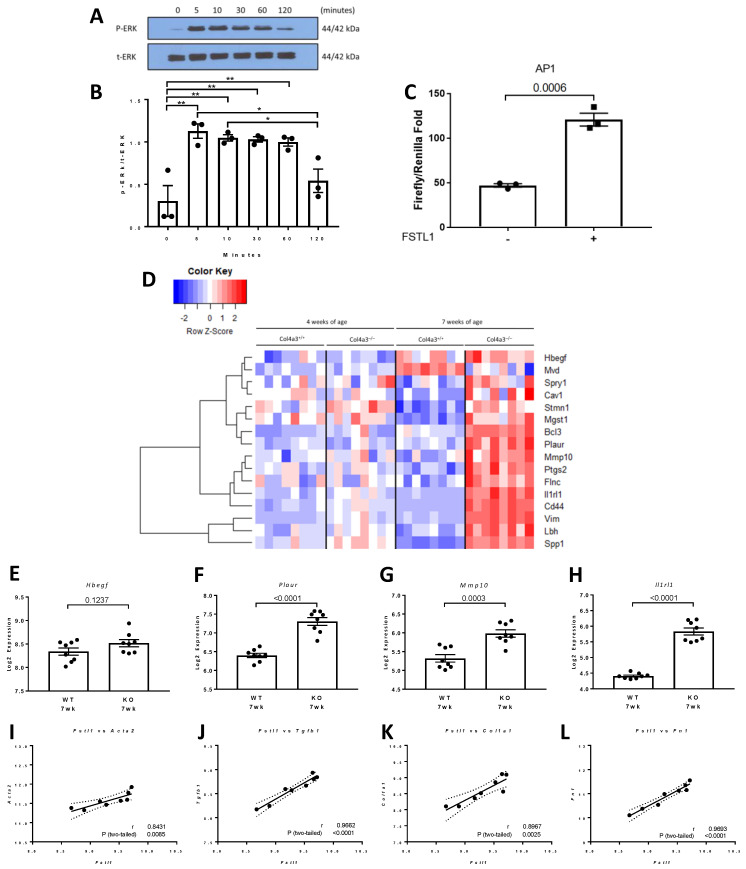
p42/p44 MAPK (ERK) activation and AP1-related gene expression. (**A**) Representative immunoblots for phosphorylated (P-ERK) and total (t-ERK) extracellular signal-regulated kinase in immortalized human proximal tubule epithelial cells that were treated with rhFSTL1 for either 0, 5, 10, 30, 60, or 120 min. (**B**) Densitometry intensities were quantified and normalized to total ERK (*n* = 3). Values are mean ± SEM. *p* values were determined by one-way ANOVA. Significance was defined as a *p* value of < 0.05. (**C**) Immortalized human proximal tubule epithelial cells were transfected with an AP1 luciferase reporter plasmid. The experimental group of cells were incubated in rhFSTL1 for 24 h (*n* = 3 per group). Luciferase activity was subsequently determined. Values are the mean ± SEM (black bars). Values are mean ± SEM, and significance was defined as a *p* value of < 0.05 by Student’s *t* tests. (**D**) Heat map with unsupervised hierarchical cluster analysis of AP1 related genes in kidneys of 4 and 7 week old *Col4a3*^-/-^ and wild-type mice (*n* = 8 per group). (**E**–**H**) Graphical representation of selected mRNA levels in 7 week old wild type versus 7 week old *Col4a3*^-/-^ mice. Values are mean ± SEM, and significance was defined as a *p* value of < 0.05 by Student’s *t* tests. (**I**–**L**) *Fstl1* mRNA levels were correlated with *Acta2*, *Tgfb1*, *Col1a1*, and *Fn1* mRNA levels. Pearson’s correlation coefficient (*r*) was determined, and two-tailed *p* values derived. Linear regression generated the line of best fit (solid lines) with 95% confidence intervals (dotted lines). Significance was defined as a *p* value < 0.05. * *p* value < 0.05. ** *p* value < 0.01.

We have previously implicated tubular epithelial cells, mainly of the proximal tubule and collecting duct, as well as interstitial fibroblasts in the progression of chronic kidney disease in *Col4a3*^-/-^ mice [5,6]. We therefore chose to study the effect of FSTL1 on p42/p44 MAPK phosphorylation and AP1-mediated gene expression in HK2 cells. Treatment with rhFSTL1 led to time-dependent phosphorylation of p42/44 MPAK (ERK) (Figure 10A,B). Biopsies from patients with fibrotic conditions, including kidney fibrosis, showed higher levels of nuclear AP1 transcription factors, while activation of AP1 can lead to interstitial fibrosis in several organs. Next, we studied the effect of rhFSTL1 on AP1 activation in kidney epithelial HK2 cells. We transfected HK2 cells with an AP1 luciferase reporter plasmid and measured promoter activity in response to rhFSTL1 stimulation. Treatment with rhFSTL1 was associated with 2 to 3-fold activation of AP1-mediated gene expression, as assessed by luciferase activity (*p* < 0.0006) (Figure 10C). Connie and coworkers defined a set of 17 genes regulated by AP1. We studied the expression of this AP1 signature in the 4 and 7 week old *Col4a3*^-/-^ and *Col4a3*^+/+^ mice. The heat map in Figure 10D illustrates the gene expression pattern. The majority of genes in the AP1 signature are up-regulated in 7 week old *Col4a3*^-/-^ mice compared to *Col4a3*^+/+^ mice, and the magnitude of the changes in a selected set of these genes (*Hbegf*, *Plaur*, *Mmp10*, and *Ilr1*) is shown in Figure 10E–H. Finally, we related *Fstl1* expression to the expression of four of these genes that are key fibrosis-associated genes, namely, alpha smooth muscle actin (*Acta2*) (*r* = 0.84, *p* < 0.0085) (Figure 10I), transforming growth factor beta 1 (*Tgfb1*) (*r* = 0.96, *p* < 0.001) (Figure 10J), collagen 1a1 (*Col1a1*) (*r* = 0.90, *p* < 0.0025) (Figure 10K), and fibronectin-1 (*Fn1*) (*r* = 0.97, *p* < 0.0001) (Figure 10L) by qPCR.

### 2.5. p38 MAPK and NFκB Signaling in Response to Fstl1

**Figure 11:**
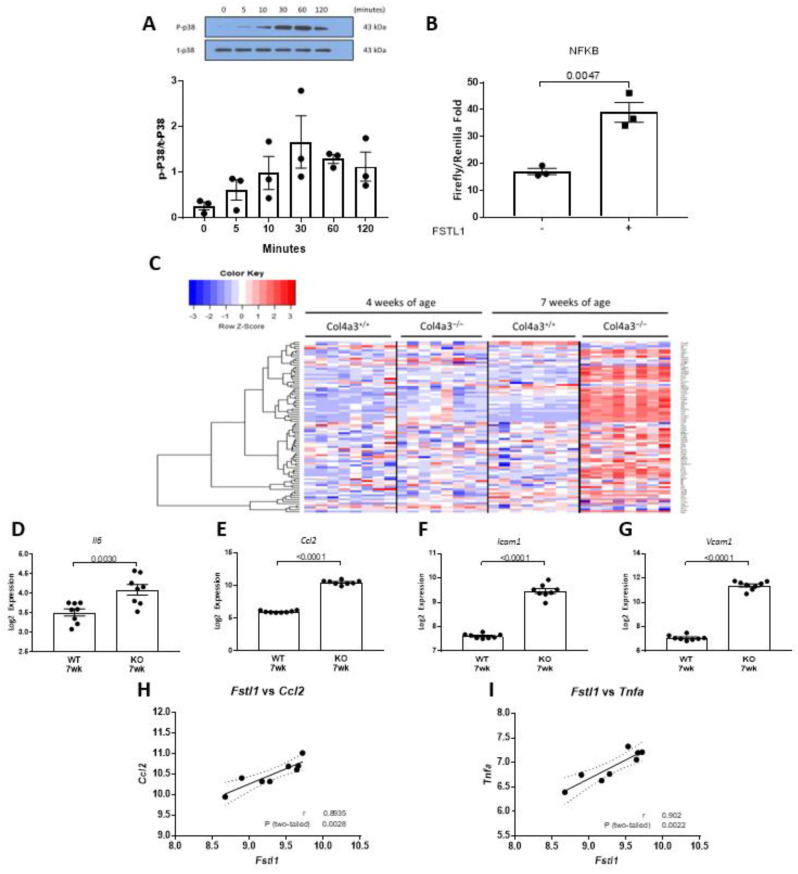
p38 MAPK activation and NFκB-related expression. (**A**) Representative immunoblots for phosphorylated (P-p38) and total (t-p38) p38 in immortalized human proximal tubule epithelial cells that were treated with rhFSTL1 for either 0, 5, 10, 30, 60, or 120 min. Densitometry intensities were quantified and normalized to total p38 (*n* = 3). Values are mean ± SEM, and *p* values were determined by one-way ANOVA. Significance was defined as a *p* value of < 0.05. (**B**) Immortalized human proximal tubule epithelial cells were transfected with an NFκB luciferase reporter plasmid. Cells were incubated in rhFSTL1 for 24 h (*n* = 3 per group) and luciferase activity was determined. Values are the mean ± SEM (black bars). Significance was defined as a *p* value of < 0.05. (**C**) Heatmap with unsupervised hierarchical cluster analysis of NFκB related genes in kidneys of 4 and 7 week old *Col4a3*^-/-^ and wild-type mice (*n* = 8 per group). (**D**–**G**) Graphical representation of selected gene mRNA levels in 7 week old wild type versus 7 week old *Col4a3*^-/-^ mice. Values are the mean ± SEM (black bars). *p* values were determined by Student’s *t* tests, and significance was defined as a *p* value < 0.05. (**H**,**I**) *Fstl1* mRNA levels were correlated with *Ccl2* and *Tnfa* mRNA levels. Pearson’s correlation coefficient (*r*) was determined, and two-tailed *p* values derived. Linear regression generated the line of best fit (solid lines) with 95% confidence intervals (dotted lines). Significance was defined as a *p* value < 0.05.

**Figure 12:**
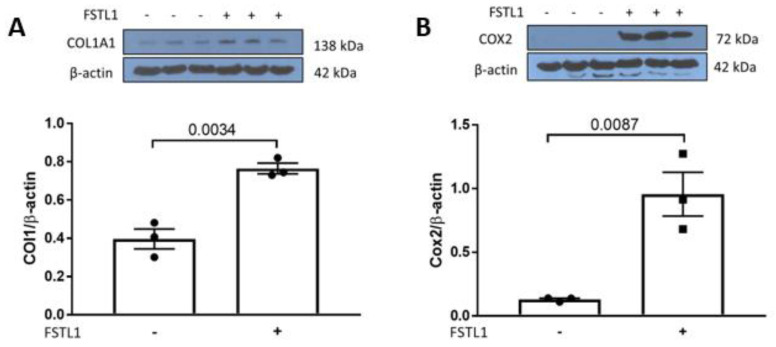
rhFSTL1 treatment of cultured human kidney cells. (**A**) Representative Western blots for collagen type I alpha1 chain (COL1a1) and β-actin in immortalized human proximal tubule epithelial cells treated with rhFSTL1 for 24 h. Densitometry intensities were quantified and normalized to total (*n* = 3). (**B**) Representative Western blots for cyclooxygenase 2 (COX2) and β-actin in immortalized human proximal tubule epithelial cells that were treated with rhFSTL1 for 24 h. Densitometry intensities were quantified and normalized to total (*n* = 3). Values are the mean ± SEM (black bars). *p* values were determined by Student’s *t* tests, and significance was defined as a *p* value < 0.05.

We have also implicated infiltrating inflammatory cells and cytokines in the progression of chronic kidney disease in *Col4a3*^-/-^ mice. Since MAPKs, including p38, promote inflammation, we explored activation of p38 by rhFSTL1 in HK2 cells. We first studied the effect of rhFSTL1 on p38 MAPK phosphorylation. Treatment with rhFSTL1 led to time-dependent phosphorylation of p38 MAPK (Figure 11A). We next studied the effect of rhFSTL1 on NFκB activation in kidney epithelial HK2 cells. We transfected HK2 cells with an NFκB luciferase reporter plasmid and measured promoter activity in response to rhFSTL1 stimulation. rhFSTL1 was associated with a 2-fold activation of NFκB-mediated gene expression, as assessed by luciferase activity (*p* < 0.0047) (Figure 11B). Pahl and coworkers assembled a list of 113 genes regulated by NFκB. We studied the expression of this NFκB signature in the 4 and 7 week old *Col4a3*^-/-^ and *Col4a3*^+/+^ mice. The heat map in Figure 11C illustrates the gene expression pattern that emerged from this unsupervised hierarchical analysis. The majority of genes in the NFκB signature are up-regulated in 7 week old *Col4a3*^-/-^ mice compared to *Col4a3*^+/+^ mice. The expression levels of *Il6* (*p =* 0.003) (Figure 11D), *Ccl2* (*p =* 0.003) (Figure 11E), *Icam1* (*p =* 0.003) (Figure 11F), and *Vcam1* (*p =* 0.003) (Figure 11G) were significantly greater in 7 week old *Col4a3*^-/-^ mice than in *Col4a3*^+/+^ mice. Finally, we related *Fstl1* expression levels to the expression of 2 of these genes that are important inflammation-associated genes, namely, monocyte chemoattractant proptein-1 or *Ccl2* (*r* = 0.89, *p* < 0.0028) (Figure 11H) and *Tnfa* (*r* = 0.90, *p* < 0.0022) (Figure 11I) by qPCR. Western blot analysis showed that rhFSTL1 increased COL1A1 (*p* = 0.0034) and COX2 protein expression in HK2 cells (*p =* 0.0087) (Figure 12B).

### 2.6. Apoptosis in Response to rhFSTL1

**Figure 13:**
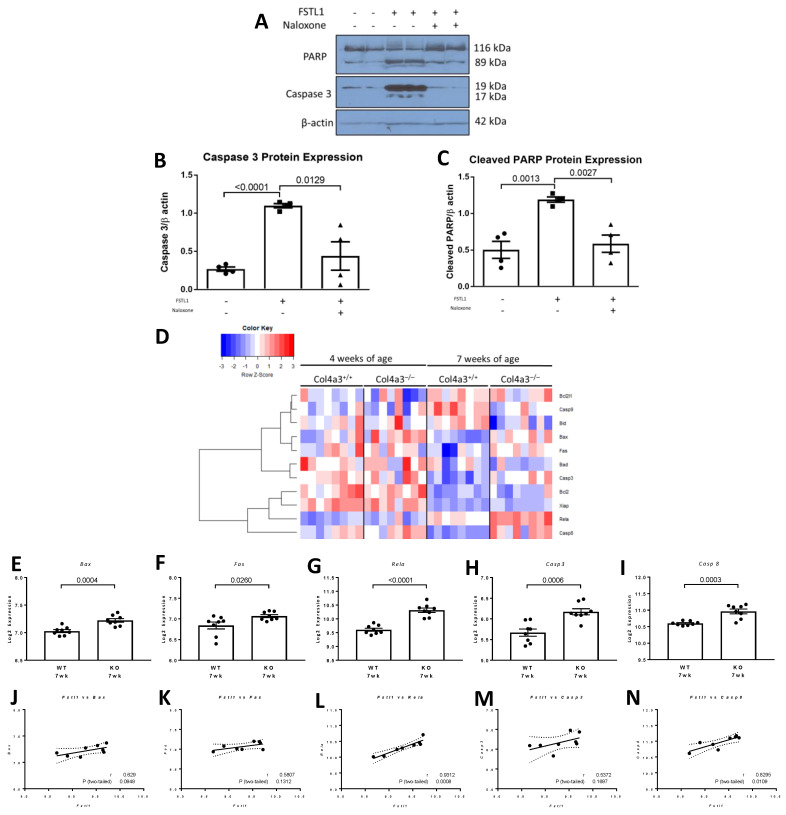
*Fstl1* apoptosis. (**A**) Representative Western blots for poly (ADP-Ribose) polymerase 1 (PARP), Caspase 3 (CASP3), and β-actin in immortalized human proximal tubule epithelial cells treated with rhFSTL1 ± naloxone for 24 h. (**B,C**) Quantitative densitometry of immunoblots for PARP and CASP3, respectively. Intensities were quantified and normalized to β-actin (*n* = 3). Values are the mean ± SEM (black bars). *p* values were determined by Student’s *t* tests, and significance was defined as a *p* value of <0.05. (**D**) Heat map with unsupervised hierarchical cluster analysis of apoptotic-related genes in kidneys of 4 and 7 week old *Col4a3*^-/-^ and wild-type mice (*n* = 8 per group). (**E**–**I**) Graphical representation of mRNA levels for selected apoptosis-related genes in 7 week old wild type versus 7 week old *Col4a3*^-/-^ mice. Values are the mean ± SEM (black bars). *p* values were determined by Student’s *t* tests, and significance was defined as a *p* value < 0.05. (**J**–**N**) *Fstl1* mRNA levels were correlated with *Bax*, *Fas*, *Rela*, *Casp3*, and *Casp8* mRNA levels in 7 week old *Col4a3*^-/-^ mice. Pearson’s correlation coefficient (*r*) was determined, and two-tailed *p* values derived. Linear regression generated the line of best fit (solid lines) with 95% confidence intervals (dotted lines). Significance was defined as a *p* value of <0.05.

Pathology studies have shown that tubular atrophy and cell loss are features of chronic kidney disease but a role for FSTL1 in apoptosis in the kidney is unknown. Accordingly, we studied the effect of rhFSTL1 on apoptosis in HK2 cells. Treatment with rhFSTL1 increased PARP cleavage and CASP3 activation, as assessed by Western blot analysis (Figure 13A). Densitometry showed a 3-fold rise in CASP3 activation (*p* < 0.0001) and a 2-fold rise in PARP cleavage (*p =* 0.0013) (Figure 13B,C, respectively). Interestingly, pre-treatment with naloxone, a TLR4 receptor antagonist attenuated the effects of rhFSTL1 on these measures of apoptosis (Figure 13B,C). We then studied the expression of 12 genes implicated in apoptosis in 4 and 7 week old *Col4a3*^-/-^ and *Col4a3*^+/+^ mice. The heat map in Figure 13C illustrates the gene expression pattern that emerged from this unsupervised hierarchical cluster analysis. There was no dominant expression pattern in the 7 week old *Col4a3*^-/-^ mice compared to 7 week old *Col4a3*^+/+^ mice. However, the expression levels of several pro-apoptotic genes including *Bax* (*p =* 0.0004) (Figure 13E), *Fas* (*p =* 0.026) (Figure 13F), *Rela* (*p* < 0.001) (Figure 13G), *Casp3* (*p* < 0.0006) (Figure 13H), and *Casp8* (*p =* 0.0003) (Figure 13I), were significantly greater in 7 week old *Col4a3*^-/-^ mice than in *Col4a3*^+/+^ mice. Finally, we related *Fstl1* expression levels to the expression of these six pro-apoptotic genes: *Bax* (*r* = 0.63, *p =* 0.0948) (Figure 13J), *Fas* (*r* = 0.58, *p =* 0.13) (Figure 13K), *Rela* (*r* = 0.93, *p* < 0.0008) (Figure 13L), *Casp3* (*r* = 0.54, *p* < 0.16) (Figure 13M), and *Casp8* (*r* = 0.83, *p =* 0.011) (Figure 13N). Although the relationships for all six genes exhibited similar trends, only two associations were statistically significant: *Rela* and *Casp8*.

### 2.7. STRING Analysis of FSTL1 Protein–Protein Interactions

**Figure 14:**
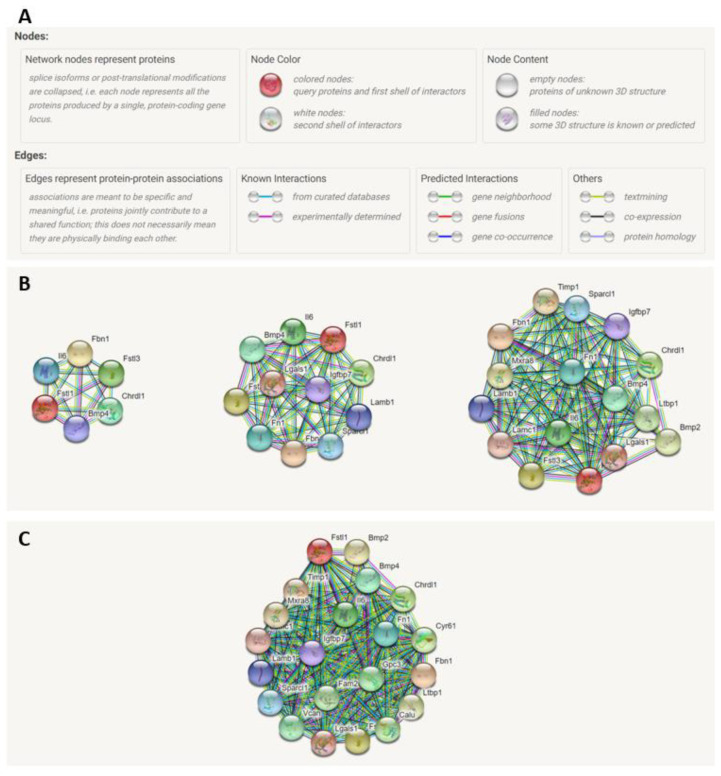
Protein–protein interaction (PPI) analysis of FSTL1. (**A**) Descriptions of nodes and edges used in the PPI interaction map. (**B**) STRING interaction map showing protein–protein association between FSTL1 and 5, 10, and 15 proteins. (**C**) STRING interaction map showing protein–protein association between FSTL1 and 20 proteins (listed in Figure 15 with the confidence scores generated by STRING).

We next used STRING analysis to generate a list of proteins that may interact with FSTL1. Figure 14B shows the network as the number of proteins and interactions increase, and colored lines show the type of interaction between two nodes or proteins. The three shells shown in Figure 14B represent a protein–protein interaction network that starts with five proteins, which is then increased to 10 proteins, and then 15 proteins. Figure 14C shows a shell with 20 FSTL1-interacting proteins. We designated the list of 20 proteins generated by this STRING analysis of interactions to be an FSTL1 signature.

**Figure 15:**
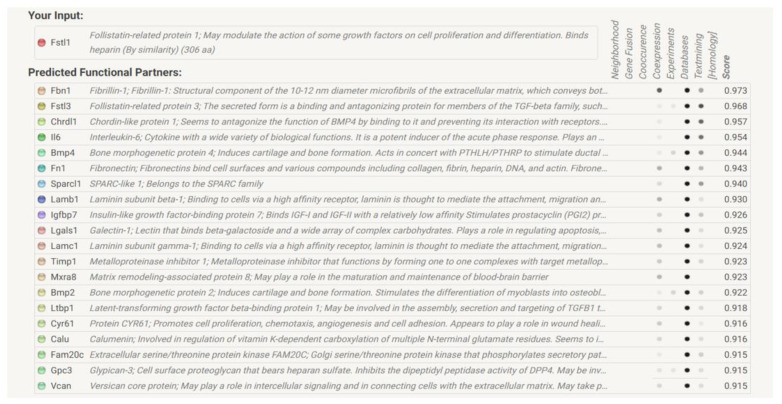
STRING interaction map showing protein–protein association between *Fstl1* and 20 proteins.

**Figure 16:**
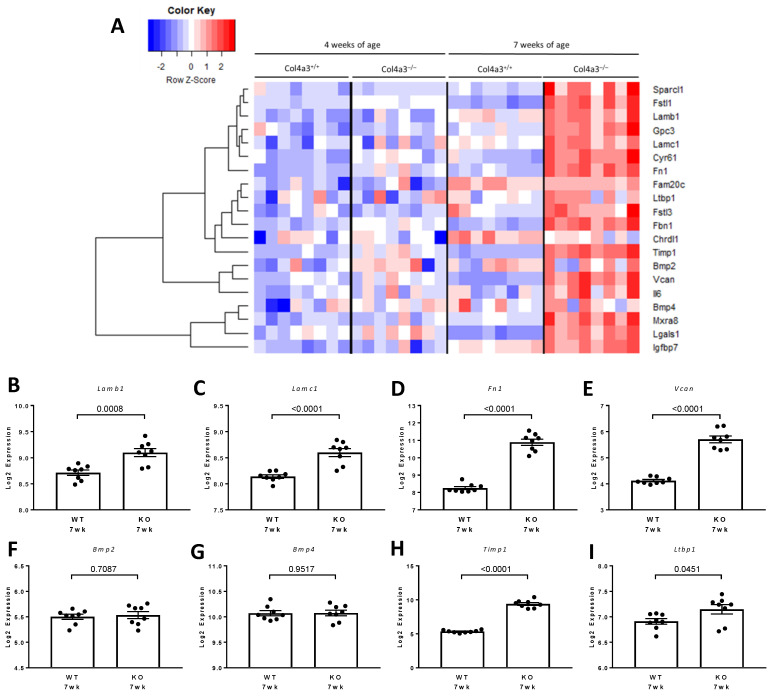
Expression analysis of FSTL1 signature genes derived from the STRING analysis of the FSTL1 protein–protein interaction (PPI) network. (**A**) Heat map with unsupervised hierarchical cluster analysis of the FSTL1 driven PPI network genes in kidneys of 4 and 7 week old *Col4a3*^-/-^ mice and wild-type mice (*n* = 8 per group). (**B**–**I**) Graphical representation of mRNA levels for selected FSTL1 signature genes in 7 week old wild type versus 7 week old *Col4a3*^-/-^ mice. Values are the mean ± SEM (black bars). *p* values were determined by Student’s *t* tests, and significance was defined as a *p* value < 0.05.

Figure 15 shows the 20 proteins comprising the largest network. This lists the protein names and provides the color code for the corresponding node (protein). We then examined mRNA levels of these proteins in microarray expression data from 4 and 7 week old *Col4a3*^-/-^ and *Col4a3*^+/+^ mice. Figure 16A illustrates the gene expression pattern that emerged from an unsupervised hierarchical analysis in the four groups. In general, most of the genes representing the FSTL1 signature were over-expressed in the 7 week old *Col4a3*^-/-^. We explored the relative expression of eight representative genes in this signature (Figure 16B–I). The expression of the extracellular proteins LAMB1 (*p =* 0.0008) (Figure 16B), LAMC1 (*p* < 0.0001) (Figure 16C), and FN1 (*p* < 0.001) (Figure 16D), and VCAN (*p* < 0.001) (Figure 16E) were all significantly increased in the 7 week old *Col4a3*^-/-^ compared to the 7 week old *Col4a3*^+/+^ mice. The expression levels of 2 bone morphogenic proteins, BMP2 and BMP4, were similar in the 7 week old *Col4a3*^-/-^ and *Col4a3*^+/+^ mice (Figure 16F,G). Interestingly, expression levels of two proteins in the FSTL1 signature that regulate the accumulation of extracellular matrix proteins, TIMP1 and LTBP1, were also increased in 7 week old *Col4a3*^-/-^ mice compared to 7 week old *Col4a3*^+/+^ mice (Figure 16H,I).

### 2.8. Studies of the Expression of Fstl1 and Its Cognate Receptors in Mice Subjected to Unilateral Ureteral Obstruction (UUO)

**Figure 17:**
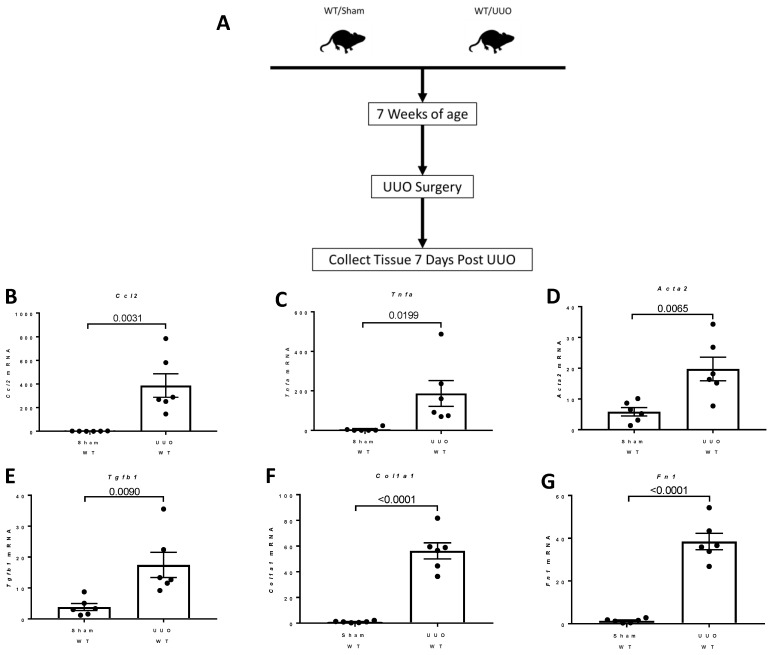
Unilateral ureteral obstruction (UUO) and *Fstl1* expression. (**A**) Schematic diagram summarizing experimental workflow and collection of tissue from 7 week old C57B6 mice subjected to sham (*n* = 4) or UUO (*n* = 5) surgery. 7 days after surgery, mice were sacrificed, and kidney tissue was collected for analysis of mRNA levels. (**B**,**C**) Graphical representation of mRNA levels for selected genes implicated in kidney inflammation (*Ccl2*, *Tnfa*) in 7 week old wild type sham versus 7 week old UUO mice. Values are mean ± SEM (black bars). *p* values were determined by Student’s *t* tests, and significance was defined as a *p* value < 0.05. (**D**–**G**) Graphical representation of mRNA levels for selected genes implicated in kidney fibrosis (*Acta2*, *Tgfb1*, *Col1a1*, *Fn1*) in 7 week old wild type sham versus 7 week old UUO mice. Values are mean ± SEM (black bars). *p* values were determined by Student’s *t* tests, and significance was a *p* value < 0.05.

**Figure 18:**
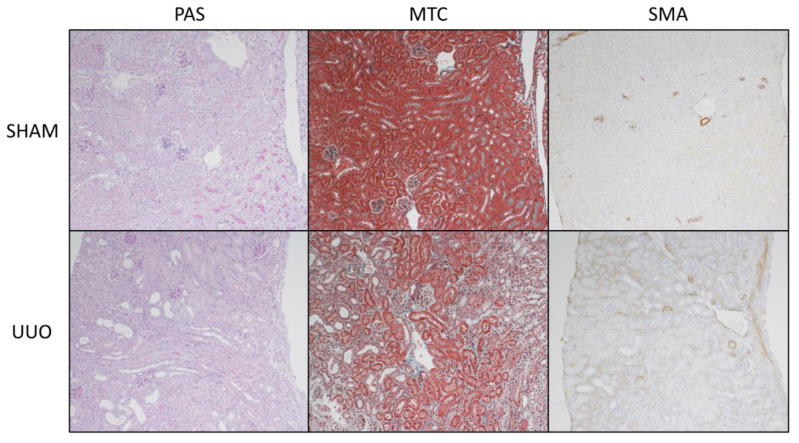
*Fstl1* Histology of sham and UUO mice. Periodic acid–Schiff (PAS) (left panels), Masson Trichrome (MTC) (middle panels), and alpha-Smooth Muscle Actin (SMA) (right panels) in sham-operated mice (upper panels) and mice subjected to UUO for 7 days (lower panels).

**Figure 19:**
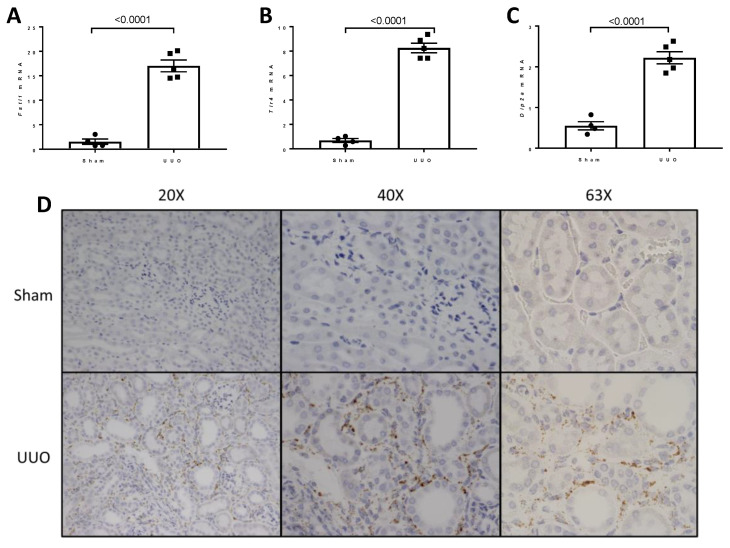
*Fstl1* expression and localization in UUO. (**A**–**C**) mRNA levels for *Fstl1* and its putative receptors (*Tlr4* and *Dip2a*) were determined by quantitative polymerase chain reaction in kidneys of C57B6 mice (sham *n* = 4, UUO *n* = 5). Values are the mean ± SEM (black bars). *p* values were determined by Student’s *t* test, and significance was defined as a *p* value of < 0.05. (**D**) Light microscopic images at three magnifications of RNASCOPE^®^ *Fstl1* localization in sham-operated mice (upper three panels) and mice subjected to UUO for 7 days (lower three panels).

UUO is associated with the rapid development of inflammation and fibrosis, and is a standard model of CKD. Figure 17A shows the experimental design. Under isoflurane anesthesia, 7 week old wild type mice were subjected to sham surgery or ligation of the left ureter (UUO). mRNA analysis and RNAscope^®^ analysis of *Fstl1* mRNA localization was performed in the left kidney after 7 days. Figure 17B–G shows the analysis of expression of genes implicated in inflammation, *Ccl2* (Figure 17B) and *Tnfa* (Figure 17C), and genes involved in fibrosis, *Acta2* (Figure 17D), *Tgfb1* (Figure 17E), *Col1a1* (Figure 17F), and *Fn1* (Figure 17G) by qPCR. As expected, the expression of this set of genes is markedly up-regulated in mice subjected to UUO compared to sham-operated mice. We characterized kidney inflammation and fibrosis in the UUO mouse in previous studies [14]. UUO recapitulates many of the cellular processes responsible for progressive kidney injury which is a commonly used model of CKD [15,16]. Figure 18 depicts light micrographic images of kidneys from sham-operated and UUO mice (7 days after surgery). Figure 19 shows the analysis of expression of *Fstl1* (Figure 19D) and the cognate receptors *Tlr4* (Figure 19B) and *Dip2a* (Figure 19C). The expression of all three genes is up-regulated in mice subjected to UUO compared to sham-operated mice at 7 days. Remarkably, the expression of *Fstl1* rose almost 10-fold (*p* < 0.0001). We then used RNAscope^®^ to localize *Fstl1* in both groups of mice. Figure 19B shows representative light micrographs at 20× and 40× magnification. It was difficult to discern any *Fstl1* expression by RNAscope^®^ in the kidneys of mice subjected to sham operation. However, in mice subjected to UUO, cells expressing *Fstl1* were present in the interstitial space, just as we had observed in the 7 week old *Col4a3*^-/-^ mice.

### 2.9. Studies of the Expression of Kidney Fstl1 Expression in the NEPTUNE Cohort

#### 2.9.1. Patient Characteristics

We studied three NEPTUNE cohorts. Table 2 shows clinical and pathologic indices of the 3 cohorts. There were 111 subjects in the focal segmental glomerulosclerosis (FSGS) cohort: 66 males and 45 females; 39 subjects in the IgA nephropathy (IgAN) cohort: 28 males and 11 females; and 61 subjects in the membranous nephropathy (MN) cohort: 39 males and 22 females (Table 2). There were missing values for some clinical and laboratory parameters, and we did not input missing values for our analyses. The average age of the FSGS group was 32.6 ± 2.0 years with a mean eGFR of 72.9 ± 3.2 mL/min/1.73 m^2^. The average age of the IgAN group was 36.1 ± 2.7 years with a mean eGFR of 67.5 ± 5.6 mL/min/1.73 m^2^. The average age of the MN group was 50.9 ± 1.8 years with a mean eGFR of 80.3 ± 3.2 mL/min/1.73 m^2^. Table 2 shows the mean values for the timed urine protein, creatinine, and albumin measures in each group. A loss of function over the course of follow-up, defined as a 40 percent decline in eGFR with an eGFR of less than 90 mL/min/1.73 m^2^, was observed in 27 subjects in the FSGS cohort, 10 subjects in the IgAN cohort, and 13 subjects in the MN cohort.

#### 2.9.2. Correlation of FSTL1 mRNA Expression with Clinical Variables

**Figure 20:**
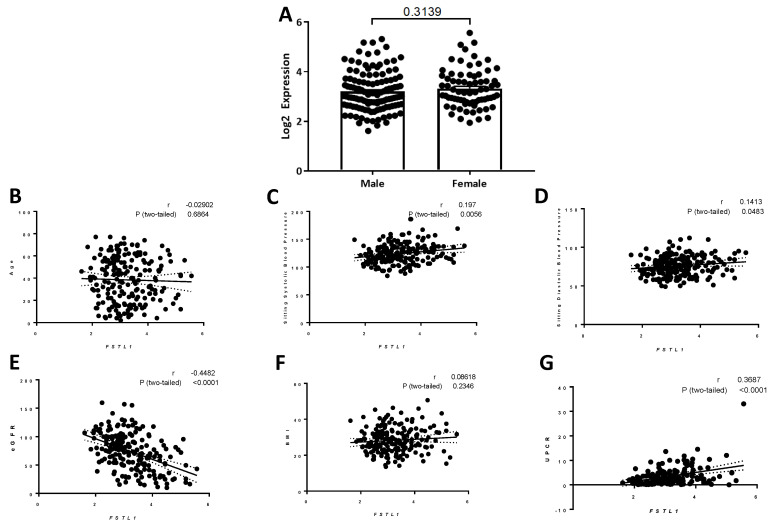
Relationship of tubulointerstitial *FSTL1* expression to clinical variables in the cohort of FSGS, IgAN, and MN. (**A**) *FSTL1* mRNA levels in male subjects compared to female subjects. (**B**–**G**) *FSTL1* mRNA levels correlated against (**B**) age, (**C**) sitting systolic blood pressure, (**D**) sitting diastolic blood pressure, (**E**) estimated glomerular filtration rate (eGFR), (**F**) Urine Protein to Creatinine Ratio (UPCR), and (**G**) Body Mass Index (BMI). Pearson’s correlation coefficient (*r*) was determined, and two-tailed *p* values derived. Significance was determined as a *p* value of <0.05. Linear regression generated the line of best fit (solid lines) with 95% confidence intervals (dotted lines). Significance was determined as a *p* value of <0.05.

We first studied *FSTL1* mRNA expression in micro-dissected kidney tubulointerstitial samples in all three cohorts as a group. Tubulointerstitial *FSTL1* mRNA expression was similar in females compared to males (*p* = 0.31; Figure 20A) and did not correlate with age (Figure 20B) or BMI (Figure 20G). There were modest correlations with sitting systolic blood pressure (*r* = 0.20, *p =* 0.0056) (Figure 20C) and sitting diastolic blood pressure (*r* = 0.14, *p =* 0.048) (Figure 20D). There was a relationship between *FSTL1* mRNA expression and eGFR (*r* = -0.49, *p* < 0.0001) (Figure 20E) such that the higher the mRNA levels for *FSTL1*, the lower the eGFR. There was also a significant relationship between centrally measured and timed UPCR values and *FSTL1* mRNA levels (Figure 20F). Multiple linear regression analysis showed that *FSTL1* expression related to age, eGFR, and UPCR but not to sex or sitting blood pressure measures (Table 3).

#### 2.9.3. FSTL1 mRNA Levels and Kidney Outcomes

**Figure 21:**
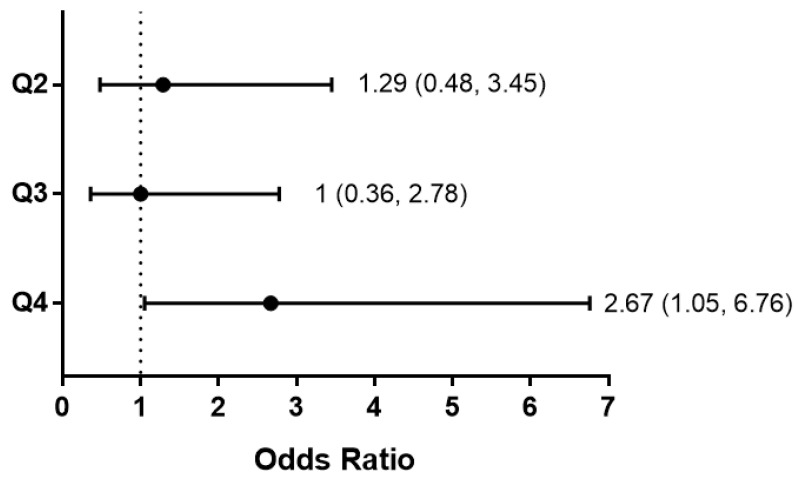
Forest Plot of End Point Analysis. The odds ratio (OR) with 95 percent confidence intervals for reaching the end point in the second, third, and fourth quartiles of baseline *FSTL1* mRNA levels. The first quartile was the reference group. The OR was not adjusted for baseline clinical variables.

Kidney disease progression was defined as a composite reaching ESKD or a 40% loss of eGFR (with a baseline eGFR<90). Table 4 shows the percent and number of individuals reaching the composite outcome for kidney disease progression in FSGS, IgAN, and MN, divided into four groups based on the quartiles for *FSTL1* mRNA levels at the time of biopsy. In the first three quartiles, 18 to 22 percent of the individuals reached the composite endpoint while 38 percent reached the composite outcome in the fourth quartile with the highest *FSTL1* mRNA levels. Figure 21 is a forest plot showing the unadjusted odds ratio and confidence intervals of reaching the composite outcome for the second to fourth quartiles compared to the first quartile (lowest *FSTL1* mRNA levels). The unadjusted odds ratio was 2.67 (1.05, 6.76) for subjects in the fourth quartile.

**Figure 22:**
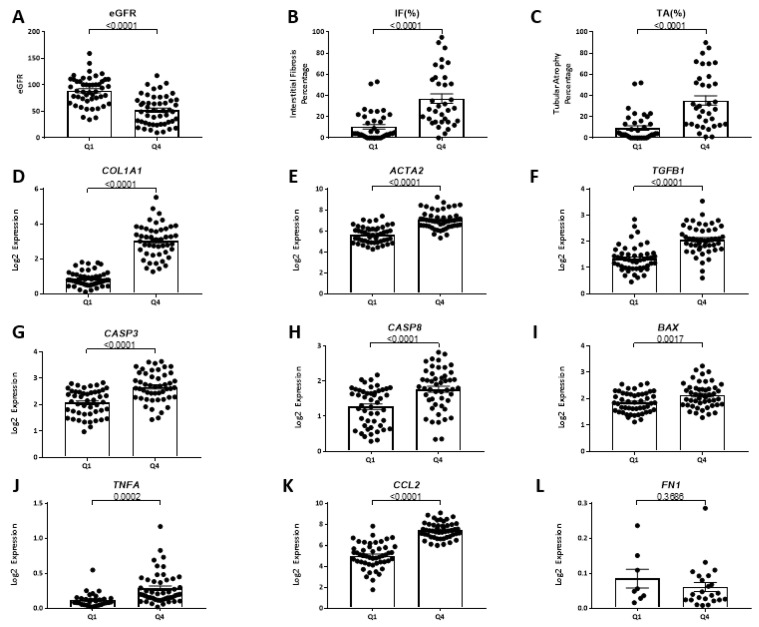
A Comparison of Baseline Laboratory Variables and Gene Expression Levels Between First and Fourth *FSTL1* Quartiles. (**A**) eGFR. (**B**) Interstitial fibrosis percentage (IF). (**C**) tubular atrophy percentage (TA). (**D**) Collagen Type I Alpha 1 Chain (*COL1A1*) expression. (**E**) Actin Alpha 2, Smooth Muscle (*ACTA2*) expression. (**F**) Transforming growth factor beta (*TGFB1*) expression. (**G**) Caspase 3 (*CASP3*) expression. (**H**) Caspase 8 (*CASP8*) expression. (**I**) BCL2 Associated X, Apoptosis Regulator (*BAX)* expression. (**J**) Tumor Necrosis Factor (*TNFA*) expression. (**K**) C-C Motif Chemokine Ligand 2 (*CCL2*) expression. (**L**) Fibronectin 1 (*FN1*) expression. Values are the mean ± SEM (black bars). *p* values were determined by Student’s *t* tests, and significance was defined as a *p* value < 0.05.

We then compared baseline measures of eGFR, interstitial fibrosis (IF), and tubular atrophy (TA) in subjects in the first and fourth quartiles for *FSTL1* mRNA levels (Figure 22). Values for eGFR were significantly lower in subjects in the fourth quartile (*p* < 0.001) (Figure 22A). Values for IF (Figure 22B) and TA (Figure 22C) were significantly higher in subjects in the fourth quartile (*p* < 0.001, and *p* < 0.001, respectively). In accordance with the measures of IF, mean mRNA levels for genes implicated in kidney fibrosis, namely, *COL1A1* (Figure 22D), *ACTA2* (Figure 22E), and *TGFB1* (Figure 22F), were greater in the fourth quartile group compared to the first quartile group (*p* < 0.001 for each mRNA). In accordance with the measures of TA, mean mRNA levels for genes implicated in apoptosis, namely, *CASP3* (Figure 22G), *CASP8* (Figure 22H), and *BAX* (Figure 22I), were greater in the fourth quartile group compared to the first quartile group (*p* < 0.001 for both *CASP3* and *CASP8*, and *p =* 0.0017 for *BAX*) (Figure 22C). There were also differences in the mean mRNA levels for genes implicated in inflammation: *TNFA* (*p =* 0.0002) (Figure 22J) and *CCL2* (*p* < 0.0001) (Figure 22K) but not for *FN1* (Figure 22L).

#### 2.9.4. FSTL1 mRNA Levels and Kidney Structure and Function

**Figure 23:**
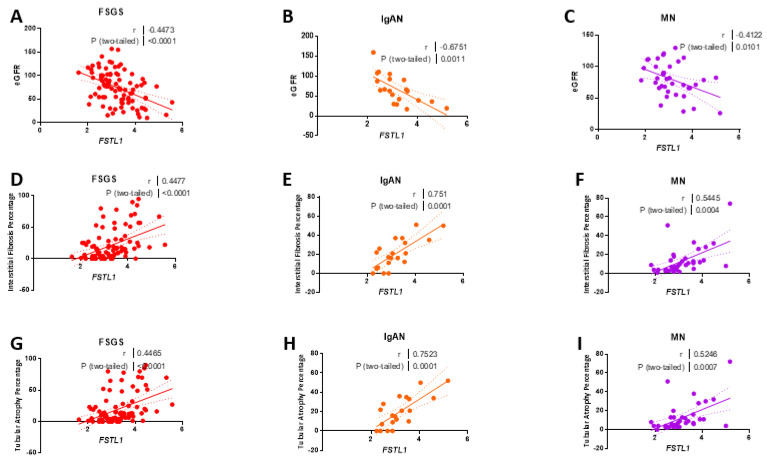
Clinical *FSTL1* expression in FSGS (red), IgAN (orange), and MN (purple). (**A**) eGFR correlated to *FSTL1* mRNA levels in FSGS patients. (**B**) eGFR correlated to *FSTL1* mRNA levels in IgAN patients. (**C**) eGFR correlated to *FSTL1* mRNA levels in MN patients. (**D**) Interstitial fibrosis (IF) correlated to *FSTL1* mRNA levels in FSGS patients. (**E**) Interstitial fibrosis (IF) correlated to *FSTL1* mRNA levels in IgAN patients. (**F**) Interstitial fibrosis (IF) correlated to *FSTL1* mRNA levels in MN patients. (**G**) Tubular atrophy (TA) correlated to *FSTL1* mRNA levels in FSGS patients. (**H**) Tubular atrophy (TA) correlated to *FSTL1* mRNA levels in IgAN patients. (**I**) Tubular atrophy (TA) correlated to *FSTL1* mRNA levels in MN patients. Pearson’s correlation coefficient (*r*) was determined, and two-tailed *p* values derived. Significance was determined as a *p* value of <0.05. Linear regression generated the line of best fit (solid lines) with 95% confidence intervals (dotted lines).

We then studied associations between eGFR, IF, TA, and *FSTL1* mRNA levels separately in each of the three cohorts. *FSTL1* mRNA levels were strongly associated with eGFR in each cohort: FSGS (*r* = −0.45, *p* < 0.0001) (Figure 23A), IgAN (*r* = −0.68, *p* < 0.0011) (Figure 23B), and MN (*r* = −0.41, *p* < 0.001) (Figure 23C). *FSTL1* mRNA levels were also strongly associated with IF in each cohort: FSGS (*r* = −0.45, *p* < 0.0001) (Figure 23D), IgAN (*r* = −0.75, *p* < 0.0001) (Figure 23E), and MN (*r* = −0.54, *p* < 0.00014) (Figure 23F). Finally, *FSTL1* mRNA levels were strongly associated with TA in each cohort: FSGS (*r* = −0.45, *p* < 0.0001) (Figure 23G), IgAN (*r* = −0.75, *p* < 0.0001) (Figure 23H), and MN (*r* = −0.53, *p* < 0.0007) (Figure 23I).

#### 2.9.5. Associations between FSTL1 mRNA Levels and Genes Implicated in Fibrosis, Inflammation, and Apoptosis, in Each of the Three Cohorts

**Figure 24:**
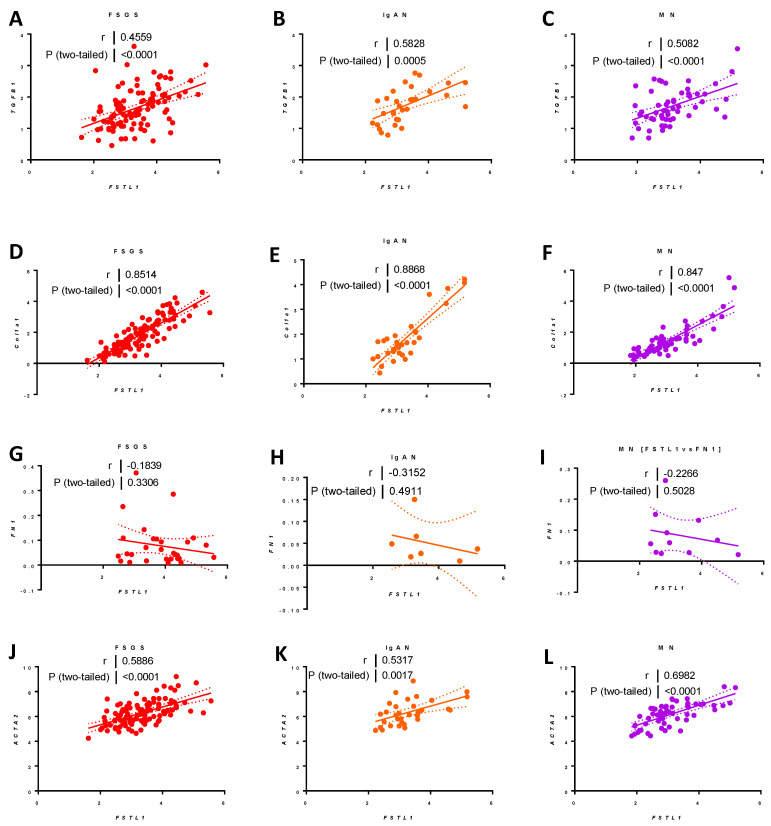
Relationship of *FSTL1* mRNA Levels to the Expression of Genes Implicated in Fibrosis in FSGS (red), IgAN (orange), and MN (purple). Table 1. expression correlated to *FSTL1* mRNA levels in FSGS (**A**), in IgAN (**B**), and MN (**C**). *COL1A1* expression correlated to *FSTL1* mRNA levels in FSGS (**D**), in IgAN (**E**), and in MN (**F**). *FN1* expression correlated to *FSTL1* mRNA levels in FSGS (**G**), in IgAN (**H**), and MN (**I**). *ACTA2* expression correlated to *FSTL1* mRNA levels in FSGS (**J**), in IgAN (**K**), and MN (**L**). Pearson’s correlation coefficient (*r*) was determined, and two-tailed *p* values derived. Significance was determined as a *p* value of <0.05. Linear regression generated the line of best fit (solid lines) with 95% confidence intervals (dotted lines).

*FSTL1* mRNA levels were strongly associated with *TGFB1* mRNA levels in each cohort: FSGS (*r* = 0.46, *p* < 0.0001) (Figure 24A), IgAN (*r* = 0.58, *p* < 0.0005) (Figure 24B), and MN (*r* = 0.51, *p* < 0.001) (Figure 24C). *FSTL1* mRNA levels were strongly associated with *COL1A1* mRNA levels: FSGS (*r* = 0.85, *p* < 0.0001) (Figure 24D), IgAN (*r* = 0.89, *p* < 0.0001) (Figure 24E), and MN (*r* = 0.85, *p* < 0.00014) (Figure 24F) and for *ACTA2* mRNA levels: FSGS (*r* = 0.59, *p* < 0.0001) (Figure 24J), IgAN (*r* = 0.53, *p* < 0.0001) (Figure 24K), and MN (*r* = 0.70, *p* < 0.00014) (Figure 24L). There was a trend that occurred for *FN1* that did not reach statistical significance (Figure 24G–I).

**Figure 25:**
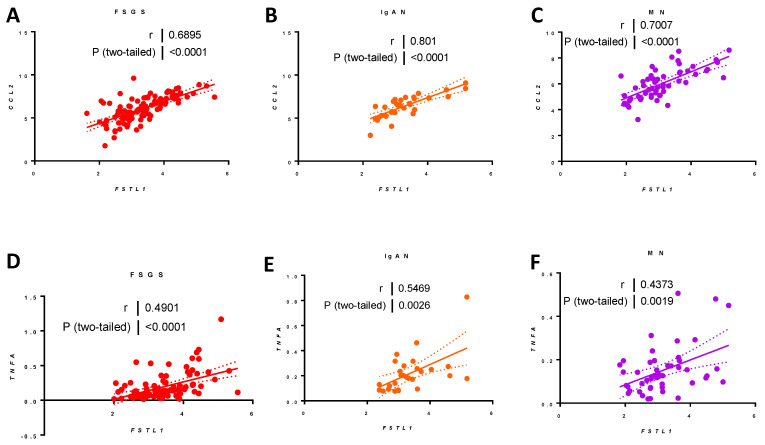
Relationship of *FSTL1* mRNA Levels to the Expression of Genes Implicated in Inflammation in FSGS (red), IgAN (orange), and MN (purple). *CCL2* expression correlated to *FSTL1* mRNA levels in FSGS (**A**), in IgAN (**B**), and MN (**C**). *TNFA* expression correlated to *FSTL1* mRNA levels in in FSGS (**D**), in IgAN (**E**), and MN (**F**). Pearson’s correlation coefficient (*r*) with two-tailed *p* values were calculated. Significance was determined as a *p* value of <0.05. Linear regression generated the line of best fit (solid lines) with 95% confidence intervals (dotted lines).

*FSTL1* mRNA levels were strongly associated with *CCL2* mRNA levels: FSGS (*r* = 0.69, *p* < 0.0001) (Figure 25A), IgAN (r = 0.80, *p* < 0.0011) (Figure 25B), and MN (*r* = 0.70, *p* < 0.001) (Figure 25C) and associated with *TNFA* mRNA levels: FSGS (r = 0.49, *p* < 0.0001) (Figure 25D), IgAN (*r* = 0.55, *p* < 0.0001) (Figure 25E), and MN (*r* = 0.44, *p* < 0.00014) (Figure 25F).

**Figure 26:**
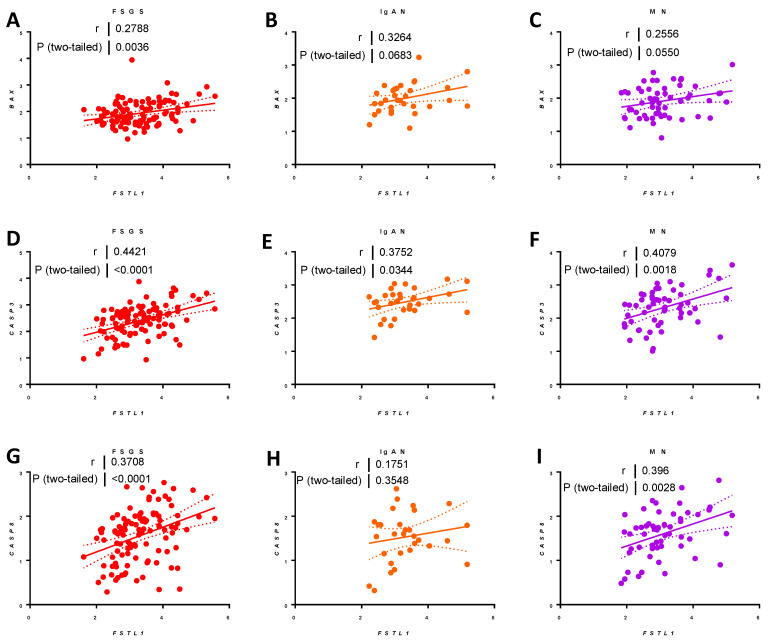
Relationship of *FSTL1* mRNA levels to the Expression of Genes implicated in Apoptosis in FSGS (red), IgAN (orange), and MN (purple). *BAX* expression correlated to *FSTL1* mRNA levels in FSGS (**A**), in IgAN (**B**), and MN (**C**). *CASP3* expression correlated to *FSTL1* mRNA levels in in FSGS (**D**), in IgAN (**E**), and MN (**F**). *CASP8* expression correlated to *FSTL1* mRNA levels in in FSGS (**G**), in IgAN (**H**), and MN (**I**). Pearson’s correlation coefficient (*r*) with two-tailed *p* values were calculated. Significance was determined as a *p* value of <0.05. Linear regression generated the line of best fit (solid lines) with 95% confidence intervals (dotted lines).

Finally, we looked at apoptosis-related genes. *FSTL1* mRNA levels were modestly associated with *BAX* mRNA levels: FSGS (*r* = 0.29, *p* < 0.0036) (Figure 26A), IgAN (*r* = 0.32, *p* < 0.06) (Figure 26B), and MN (*r* = 0.26, *p* < 0.06) (Figure 26C). *FSTL1* mRNA levels were more strongly associated with *CASP3* mRNA levels: FSGS (*r* = 0.44, *p* < 0.0001) (Figure 26D), IgAN (*r* = 0.38, *p* < 0.03) (Figure 26E), and MN (*r* = 0.41, *p* < 0.0018) (Figure 26F), and *CASP8* mRNA levels in FSGS (*r* = 0.37, *p* < 0.0001) (Figure 26G) and MN (*r* = 0.40, *p* < 0.0028) (Figure 26I). There was a trend that occurred for *CASP8* that did not reach statistical significance in IgAN (Figure 26H).

## 3. Discussion

There is still a limited understanding of the mechanism(s) responsible for the progression of CKD [17]. Here, we present an experimental design and microarray-based approach to identify new genes that may play a role in the progression of tubule-interstitial (TI) injury. We utilized *Col4a3*^-/-^ mice because they develop early proteinuria followed by TI inflammation and fibrosis leading to mortality by 9–10 weeks of age [5,7]. Proteinuria is an important risk factor for loss of kidney function in human kidney disease [18]. Although developed as a model of Alport Syndrome, studies of *Col4a3*^-/-^ mice may also provide insights into the pathogenesis of a wider spectrum of kidney disease because the *Col4a3* gene plays a role in both sporadic and familial FSGS as well as diabetic nephropathy [19,20]. Moreover, changes in the TI of the kidney, in particular fibrosis and tubular atrophy, correlate inversely with GFR [21,22].

We first conducted an analysis of differential gene expression in 4 week old and 7 week old *Col4a3*^+/+^ mice and *Col4a3*^-/-^ mice. Albuminuria is already elevated at 4 weeks of age, but as we have previously reported, there is little focal glomerulosclerosis, interstitial fibrosis, or inflammation evident in light microscopy. Electronic microscopy will show focal thinning and splitting of the GBM [5,7]. Serum creatinine levels are similar in 4 week old *Col4a3*^+/+^ mice and 4 week old *Col4a3*^-/-^ mice so that GFR has not yet declined. As expected, *Col4a3*^-/-^ mice already exhibit higher levels of expression of the mature collagens, *Col4a3*, *Col4a4* than *Col4a5*, at 4 weeks of age, compared to the immature collagens, *Col4a1* and *Col4a2*. This developmental switch in collagen gene expression does not occur in *Col4a3*^-/-^ mice, and the maintenance of expression of *Col4a1* and *Col4a2* is especially evident at 7 weeks of age. This abnormal gene expression pattern is associated with even higher albumin excretion rates and a rise in serum creatinine in the *Col4a3*^-/-^ mice [7].

Our first major observation was that only four genes, apart from *Col4a3*, were differentially expressed in the kidneys of 4 week old *Col4a3*^+/+^ mice compared to 4 week old *Col4a3*^-/-^. *Zfp747* was the only other down-regulated gene. *Zfp747* is one of approximately 350 genes in the family of Kruppell-associated box (KRAB) domain-containing zinc finger proteins. The function of this protein is unknown although other family members have been implicated in the regulation of gene transcription and DNA methylation. It has not been studied in kidney disease. The gene *Mfap4* encoding microfibril associated protein 4 was up-regulated at 4 weeks of age. This extracellular matrix protein binds to collagens, elastin, and fibrillin-1, and it is a paralog of fibronectin. Recent studies have linked *Mfap4* to liver fibrosis, cardiac dysfunction, and kidney fibrosis [23,24,25]. The gene *Cald1* encodes caldesmin-1, an intracellular protein that regulates cell contraction and interacts with the actin cytoskeleton. Interestingly, *Cald1* mRNA levels are also elevated in the glomeruli of humans with diabetic nephropathy [26]. There was a sustained increase in expression of both *Mfap4* and *Cald1* in the kidneys of 7 week old *Col4a3*^-/-^ mice.

*Fstl1* was the third gene in which the mRNA levels were elevated at 4 weeks of age, and we chose to focus our studies on *Fstl1* because it is a secreted extracellular protein known to be a ligand for Toll-like receptors [27]. *Fstl1* has been linked to pulmonary fibrosis and inflammation, particularly in experimental arthropathies [28,29]. *Fstl1* may play a role in cisplatin-induced acute kidney injury [30]. Studies in a remnant kidney model of CKD suggested that it functioned as an endocrine factor produced by the heart, secreted, and then delivered to the kidney by renal blood flow [30]. In contrast, the Protein Atlas suggested that it was endogenously expressed in kidney tubular epithelial cells, and studies suggested that in UUO kidneys, *Fstl1* was expressed in cells of the loop of Henle and/or collecting duct [31]. Accordingly, our next major finding was that *Fstl1* is expressed in cells in the interstitial compartment of the kidney. Our RNAScope© analysis also showed that there is very little expression in normal kidneys. Single cell transcriptomic data localized expression to fibroblasts/myofibroblasts and not in tubular epithelial cells. We previously described cell populations contributing to progression signature gene expression, and one of the important themes that emerged from this work was that fibroblasts contributed to CKD progression in *Col4a3*^-/-^ mice [6]. Taken together, these findings show that *Fstl1* is fibroblast-derived.

To further explore the potential paracrine role of *Fstl1* in the kidney we next looked at the expression of putative *Fstl1* receptors in the kidney: three receptors have been identified. The first is Toll-like receptor-four (*Tlr4*) [27]. *Tlr4* is expressed in tubular epithelial cells as well as in infiltrating monocytes/macrophages Leucocytes, including macrophages, and renal epithelial cells express the *Tlr4* receptor, and it does play a role in kidney injury [32]. Zhang and coworkers showed that deletion of the gene for *Tlr4* attenuated cisplatin-induced kidney injury. In order to determine if the effect is due to loss of *Tlr4* in myeloid cells or loss of *Tlr4* in renal tubular cells the investigators generated bone marrow chimeric mice. They found that the protective effect of *Tlr4* gene deletion was due to loss of expression in kidney tubular cells [32]. This work supports our notion that FSTL1 could function as a paracrine factor in the kidney to affect the progression of chronic kidney disease by engaging *Tlr4* receptors on kidney tubular cells. *Cd14* is also linked to the ligand-like function of *Fstl1*, and it is predominantly expressed in monocytes/macrophages [27]. Interestingly tubular epithelial expression of *Cd14* is up-regulated in murine models of kidney injury [33]. Disco Interacting Protein 2 Homolog A (*Dip2a*) is the third receptor linked to *Fstl1* cell signaling but little is known about the intracellular pathways activated by the receptor [34]. Vascular tissue including the kidney expresses *Dip2a* [12,34]. We next found that both *Tlr4* and *Cd14* were up-regulated in *Col4a3*^-/-^ mice. *Tlr4* expression was increased at 7 weeks of age while *Cd14* was already increased at 4 weeks of age in *Col4a3*^-/-^ mice compared to *Col4a3*^+/+^ mice. These findings support the hypothesis that activation of this ligand/receptor pathway increases in *Col4a3*^-/-^ mice and thus *Fstl1* can readily function in a paracrine manner in the kidney.

To test this hypothesis in vitro, we studied the effect of rhFSTL1 on cultured kidney epithelial cells and focused on three biological processes important in chronic kidney disease pathogenesis: fibrosis, inflammation, and apoptosis. *AP1*-mediated gene expression is postulated to play a key role in the regulation of gene expression related to fibrosis [35]. rhFSTL1 activates ERK in a time-dependent manner, and *AP1* is downstream of ERK activation. rhFSTL1 also activated *AP1*-mediated gene expression in kidney tubular cells based on the activation of an *AP1* promoter construct that drove firefly luciferase expression. It is possible that fibroblast derived FSTL1 could function in a paracrine manner to activate canonical MAPK (ERK) signaling in tubular cells converging on *AP1*. We then compared the expression levels of a defined set of genes that are regulated by *AP1* in 4 and 7 week old *Col4a3*^-/-^ mice compared to *Col4a3*^+/+^ mice. There was a marked increase in the expression of the majority of the *AP1*-mediated genes including plasminogen activator, urokinase receptor (*Plaur*), matrix metalloproteinase 10 (*Mmp10*), and interleukin1 receptor-like1 (*Il1rl1*). *Plaur* promotes plasmin formation and plays a role in the regulation of extracellular matrix protein degradation as well as the activation of growth factors including *TGFB1*. *Mmp10* also regulates extracellular matrix protein degradation [36]. Taken together, the changes in the expression of these two genes emphasize the important role of extracellular matrix remodeling in the progression of fibrosis in the kidneys of *Col4a3*^-/-^ mice. *Il1rl1* is a member of the interleukin 1 receptor family, and it may be pro-inflammatory linking *AP1*-regulated gene expression to inflammation in the kidney [37]. Although it is very unlikely that FSTL1 is the only ligand contributing to the activation of these genes in vivo, we also saw strong correlations between *Fstl1* mRNA levels and the mRNA levels of alpha smooth muscle actin (*Acta2*), transforming growth factor beta (*Tgfb1*), *Col1a1*, and *Fn1*. Indeed, over 80–90 percent of the variability in the levels of these genes is associated with the variability in *Fstl1*. Western blot analysis showed that rhFSTL1 led to an increase in COL1A1 protein expression.

We then studied the effect of rhFSTL1 on cultured kidney epithelial cells and focused on inflammation. *NFκB*-mediated gene expression is postulated to play a key role in the regulation of gene expression related to inflammation [38]. One of the canonical MAPKs, p38, is classically upstream of *NFκB*. rhFSTL1 activates p38 in a time-dependent manner, and *NFκB* is downstream of ERK activation [39]. rhFSTL1 also activated *NFκB*-mediated gene expression in kidney tubular cells based on the activation of an *NFκB* promoter construct that also drove firefly luciferase expression. It is therefore possible that fibroblast-derived FSTL1 could function in a paracrine manner to activate canonical MAPK (p38) signaling in tubular cells converging on *NFκB*. We next compared the expression levels of a defined set of genes that are regulated by *NFκB* in 4 and 7 week old *Col4a3*^-/-^ mice compared to *Col4a3*^+/+^ mice. There were marked increases in expression in most of the *NFκB* -mediated genes including *Il6*, *Ccl2*, *Icam1*, and *Vcam1*. The latter three genes play a role in the recruitment of inflammatory cells including monocytes/macrophages to the kidney [40,41]. These observations are consistent with our recent report that a progression gene signature in *Col4a3*^-/-^ mice reflected at least in part, increased infiltrating inflammatory cells [6]. Again, FSTL1 is not the only ligand to increase expression of these genes in vivo, but we were able to see strong correlations between *Fstl1* mRNA levels and the mRNA levels of *Ccl2* and *Tnfa*, two cytokines implicated in the progression of chronic kidney disease [42]. Almost 90 percent of the variability in the levels of these genes relates to the variability in *Fstl1*, based on Spearman correlation analysis. Western blot analysis showed that rhFSTL1 led to an increase in COX2 protein expression.

Finally, we then studied the effect of rhFSTL1 on cultured kidney epithelial cells and focused on apoptosis because tubular atrophy is a common finding in chronic kidney disease [43]. rhFSTL1 treatment led to PARP cleavage and CASP3 activation in kidney tubular cells in vitro. Interestingly, this effect was downstream of TLR4 because pre-treatment with the TLR4 receptor antagonist, naloxone, abrogated the effect. Fibroblast derived FSTL1 could function in a paracrine manner to induce apoptosis in adjacent kidney tubular cells and contribute to the loss of kidney tubular cell mass or tubular atrophy. We next compared expression levels of a set of genes that play a role in apoptosis in 4 and 7 week old *Col4a3*^-/-^ mice compared to *Col4a3*^+/+^ mice. There was a marked increase in expression in most of the apoptosis genes, including *Bax*, *Fas*, *Rela*, *Casp3*, and *Casp8*, although it is again likely that many different ligands influence the expression of these genes in vivo. We were able to see strong correlations between *Fstl1* mRNA levels and the mRNA levels of *Bax*, *Rela*, and *Casp8*. *Fstl1* accounted for between 60 and 90 percent of the variability in the levels of these genes, based on Spearman correlation analysis.

Taken together, these in vivo and in vitro findings suggest that FSTL1 promotes the progression of chronic kidney disease in *Col4a3*^-/-^ mice and that it functions in a paracrine manner to influence the biology of tubular cells and infiltrating monocytes/macrophages, although we did not examine the latter directly. Adams and coworkers studied the role of FSTL1 in acute kidney injury secondary to cisplatin and they suggested that FSTL1 was protective [30]. They utilized a mouse with a hypomorphic *Fstl1* gene and found that there was less inflammation in the hypomorphic mice and more kidney injury after administration of cisplain. This is interesting because they examined mRNA levels of *Il6* and *Tnfa*. They assessed tubular injury by measuring *Kim1*, and these findings suggest that the role of FSTL1 in kidney injury may be context specific [30]. Our finding of the localization of *Fstl1* in the interstitial compartment of the kidney is the first study to employ an in situ hybridization-like technique. Antibody-based studies have previously localized FSTL1 to the loop of Henle [30,44], but RNAScope© is not dependent on antibody specificity or confounded by kidney cell auto-fluorescence. This may account for our novel finding. Single cell transcriptomic data from human kidneys confirmed that *Fstl1* was restricted to the interstitial compartment and localized in fibroblasts and activated myofibroblasts. Moreover, studies of cutaneous wound healing strongly implicated FSTL1 in scar formation, and localized its expression to fibroblasts, in accordance with our work [45,46].

A novel feature of our studies was the definition of an *Fstl1* gene signature. In order to perform an unbiased assessment of the potential role of *Fstl1* in the progression of CKD in *Col4a3*^-/-^ mice we completed an in silico analysis of FSTL1 protein–protein interactions using STRING analysis. This analysis was independent of our studies of gene expression, and it generated a novel network of proteins linked to FSTL1 based on evidence in the experimental literature—in a sense, an FSTL1 signature. We created four networks consisting of 5 to 20 proteins by limiting the output of the number of interacting proteins. A colored node represents each protein in the network. The color of the edge connecting two nodes indicates the type of interaction. For example, “experimentally derived” data linking FSTL1 to another protein is a purple edge while “gene co-occurrence” between FSTL1 and another protein is a dark blue edge.

In STRING, each protein–protein interaction generates scores that in combination yield a final interaction score. The interaction score does not indicate the strength or even the specificity of the protein–protein interaction, but the final score does indicate the confidence or likelihood that an interaction is true given the evidence used to calculate the score. The scores can range from 0 to 1. The higher the score the greater the confidence [47]. Scores for the individual proteins in the FSTL1 interaction network are shown in the last column of Figure 15, Panel A and range from 0.915–0.973, suggesting that the analysis yielded proteins with a high likelihood of interacting with FSTL1.

We then generated a heat map based on the expression levels of the genes for the proteins in the 20 protein STRING diagram. Several of the genes in this list of proteins were over-expressed in the 7 week old *Col4a3*^-/-^ mice compared to the 7 week old Col4a^+/+^ mice as illustrated in the heat map. A number of extracellular matrix proteins are part of this FSTL1 signature, including laminins (LAMB1and LAMC) and fibronectin (FN1) along with extracellular proteins that regulate matrix protein turnover. This group included the tissue inhibitor of metalloproteinase-one (TIMP1) and Latent Transforming Growth Factor Beta Binding Protein-one (LTBP1), the latter a protein involved in the activity of TGFB1, a pro-fibrotic cytokine. Bone morphogenic proteins were also part of the STRING network (BMP2 and BMP4). Together with our observations on *AP1*, the STRING analysis also relates FSTL1 to re-modeling of the extracellular matrix and the development of interstitial fibrosis, a critical event that is characteristic of progressive CKD [48].

We extended our studies of *Fstl1* and its receptors *Tlr4* and *Dip2a* in another experimental model of chronic kidney disease: murine UUO. This model of kidney injury is associated with marked increases in the expression of genes involved in kidney fibrosis (*Acta2*, *Tgfb1*, *Col1a1*, and *Fn1*) and inflammation (*Ccl2* and *Tnfa*) 7 days after ligation of the ureter. There was a 10-fold increase in *Fstl1* and an 8-fold increase in *Tlr4* expression after 7 days. *Dip2a* also increased 4-fold. An RNAScope© analysis also showed that there is very little expression in the normal kidney and that the increase in *Fstl1* in the UUO kidney occurs in the interstitial compartment, as we had observed in the *Col4a3*^-/-^ mice. *Fstl1* and its cognate receptors are therefore up-regulated in two different models of experimental CKD, one associated with early glomerular injury and progressive proteinuria and another associated with urinary tract obstruction. These observations spurred us to extend our analysis of *Col4a3*^-/-^ mice to human CKD.

Our studies of human CKD involved subjects recruited to the NEPTUNE consortium study of proteinuric CKD with three common kidney diseases: focal segmental glomerulosclerosis (FSGS), IgA nephropathy (IgAN) and membranous nephropathy (MGN). We examined these diseases because each is a glomerular disease process characterized by progressive injury, declining function, and proteinuria. Interestingly, *COL4A* mutations have emerged in patients with CKD beyond Alport syndrome, including FSGS, increasing the relevance of our studies in *Col4a3*^-/-^ mice [19]. There are several strengths related to the use of this cohort of patients. First, extensive clinical data and long-term follow-up of kidney outcomes are available. Kidney biopsy samples were microdissected at baseline and gene expression profiles were derived from both the glomerular and tubule-interstitial compartments. In addition, kidney biopsy samples were subjected to protocol assessment of kidney injury including tubulo-interstitial fibrosis (IF) and tubular atrophy (TA). These experimental protocols allow for the study of gene expression and the relationship of gene expression to both clinical variables and pathological assessment of the kidney biopsy. Finally, the protocol-driven collection of longitudinal clinical data allows for the study of relationships between gene expression and kidney outcomes.

We first studied the three cohorts as a single group to relate *FSTL1* expression to clinical variables like age, sex, BMI, blood pressure, eGFR, and urinary protein excretion (UPCR). We chose to look at sex because it is an important determinant of kidney outcomes while BMI and blood pressure relate to poor kidney outcomes. We first observed that *FSTL1* expression in the kidney tubule-interstitial compartment was similar in males than females at baseline (recruitment to NEPTUNE). Univariate analysis of *FSTL1* mRNA levels and age, BMI, blood pressure (both sitting systolic and diastolic pressure) showed no significant relationships between these variables and *FSTL1* mRNA levels. In contrast, there was a significant inverse relationship between eGFR and *FSTL1* mRNA levels such that the higher the *FSTL1* expression, the lower the eGFR value at recruitment to NEPTUNE. Interestingly, we observed a positive relationship between the UPCR. This suggests that proteinuria may be an important determinant of *FSTL1* expression, although we did not define a causal relationship. Given the above findings, we performed a multiple linear regression analysis in which we related *FSTL1* expression to age, sex, BPSS, BPSD, eGFR, and UPCR. Age, eGFR, and UPCR were associated with *FSTL1* expression in the three groups of subjects as a whole.

In an unadjusted analysis of the relationship between *FSTL1* mRNA quartiles and the composite clinical outcome of ESKD or a 40% loss of kidney function, we found that the odds ratio of reaching this composite was 2.67 (1.05, 6.76) in the highest quartile of *FSTL1* mRNA levels compared to the first, second, and third quartiles. We compared mean values for eGFR, interstitial fibrosis (IF), and tubular atrophy (TA) between the first and fourth quartile for *FSTL1* mRNA levels and mean values for eGFR were significantly lower in the fourth quartile compared to the first quartile while mean values for IF and TA were greater. In accord with the increase in IF mean values for the mRNA levels of *COL1A1*, *ACTA2*, and *TGFB1* were all increased in the fourth quartile compared to the first quartile. In a similar manner, genes for the apoptosis proteins, *CASP3* and *CASP8* were higher in the fourth quartile like the measures of TA. There was also an increase in genes related to inflammation (*CCL2* and *TNFA*). Taken together, high *FSTL1* mRNA levels in the kidney identify subjects with lower eGFR, more chronic kidney injury based on IF and TA, and higher levels of genes implicated in fibrosis, inflammation, and apoptosis, cellular processes responsible for progressive loss of function.

We next divided the NEPTUNE cohort into the three groups based on underlying pathological diagnosis and looked at the correlation between *FSTL1* mRNA levels and kidney function IF, and TA in each cohort separately. The relationships in all three groups re-capitulated the analysis of the whole group. There were positive associations between *FSTL1* levels and genes involved in fibrosis and apoptosis that were similar in all three cohorts. Once again, these relationships do not establish causality but together strengthen the hypothesis that kidney expression of *FSTL1* contributes to progressive loss of function, kidney fibrosis, and loss of functioning nephron mass, and taken together with our in vitro observations do not support the hypothesis that *FSTL1* limits kidney injury. Moreover, changes in kidney expression of *FSTL1* are important and it is tempting to speculate that paracrine effects of fibroblast-derived *FSTL1* are responsible for the relationships that we have identified in experimental and clinical CKD.

An important limitation of the current study is that we did not establish the mechanism(s) responsible for the early and sustained increase in kidney *FSTL1* expression in *Col4a3*^-/-^ mice. Sundaram and coworkers were studying the role of *FSTL1* keratinocytes in chronic wound healing [45]. *FSTL1* promotes wound healing by virtue of its effects on fibrosis, analogous to the role we think that it plays in chronic kidney disease [45]. Chronic fibrotic disease may be wound healing gone awry, at least in part, as first articulated by Wayne Border in a review on *TGFB1* [49]. Sundaram discovered that a post-transcriptional switch regulated expression of *FSTL1*. This switch is due to microRNA-198 (miR-198) encoded in the 3′-umtranslated *FSTL1* transcript. A protein called KH-type splicing regulatory protein (KHSRP) influences the processing of the *FSTL1* mRNA transcript and decreases translation to the mature protein. *TGFB1* reduces the expression of KHSRP and increases the translation of *FSTL1* [45].

There was no increase in *Tgfb1* mRNA levels in the 4 week old *Col4a3*^-/-^ mice, but activation of TGFB1 is independent of transcript levels so it may still promote early increases in FSTL1 if there is a release of mature TGFB1 from its latent complex [50]. Increased reactive oxygen species can activate TGFB1 and the binding and internalization of albumin by proximal tubule cells generate superoxides [50,51]. Such an effect could contribute to early TGFB1 activation in the kidney interstitial space in *Col4a3*^-/-^ mice. There was a three-fold rise in albuminuria in the 4-week-old *Col4a3*^-/-^ mice compared to the 4-week-old *Col4a3*^+/+^ mice. This mechanism cannot account for the rise in *Fstl1* in the UUO mice because this model of kidney injury is not associated with albuminuria. However, UUO is associated with increased oxidative stress likely due to mechanical strain on the tubular epithelial cells. Moreover, we did see increases in *Tgfb1* mRNA levels in the UUO mice.

Although increased oxidative stress may be a common pathway leading to TGFB1 activation and a subsequent increase in FSTL1 in the kidney, it is also possible that activation of the renin angiotensin system contributes to TGFB1 activation. Border and coworkers reported that angiotensin II also activates TGFB1 [52]. Angiotensin II generation increases in both the *Col4a3*^-/-^ mice and in mice subjected to UUO [53]. Overall, the contribution of these processes to the increase in FSTL1 occurring in the kidney will require future study.

Our study has other limitations. We did not perform any immunohistochemistry of FSTL1 or any dual labeling immunohistochemistry to improve localization to particular kidney segments. The utilization of a kidney single cell transcriptional dataset along with RNAscope^®^ in our experimental model allowed us to take an independent approach to the localization of FSTL1. Dual labeling is thus a future goal so that we may gain a better understating of the kidney cells responsible for the secretion of FSTL1.

We did not study the impact of deletion of the gene for *Fstl1* in either the *Col4a3*^-/-^ mice or in the mice subjected to UUO. Deletion of the gene for *Fstl1* is neonatal lethal. This has limited past approaches to the use of hypomorphic alleles. The generation of a fibroblast-specific *Fstl1* gene knockout mouse is the most definitive approach to defining the effect of *Fstl1* on progressive fibrosis, but the generation of a mouse that also has *Col4a3* gene deletion would require a complex breeding strategy. Subjecting transgenic mice to UUO would be more straightforward with the caveat that this mouse model is not associated with proteinuria. Moreover, future work will be required to determine kidney tissue concentrations of *FSTL1* to better support in vitro studies of the effect of *FSTL1* on kidney cells and mononuclear cells, including studies on cell proliferation. Another important limitation of our report is the absence of an attempt to block *FSTL1* activity. Neutralizing antibodies have blocked *FSTL1* bioactivity in models of lung injury [54] but the specific reagents used by these investigators are not commercially available. Finally, our analysis of the relationship between *FSTL1* levels and kidney outcomes in the NEPTUNE cohort was unadjusted. Therefore, our analysis is exploratory, hypothesis-generating, and meant to support a more definitive future analysis using Kaplan–Meier (K-M) survival curve and Cox proportional-hazards modeling. The predictive value of *FSTL1* remains to be determined. Finally, we did not relate urinary FSTL1 to expression in the kidney and to interstitial fibrosis in the kidney. This analysis would help determine if urinary FSTL1 might be a non-invasive marker of kidney fibrosis.

In conclusion, our studies show that FSTL1 is a fibroblast-derived cytokine expressed in the kidney and up-regulated in both experimental and clinical chronic kidney disease. Our in vivo, in vitro, and in silico analyses suggest that FSTL1 contributes to fibrosis, inflammation, and apoptosis in the kidney. FSTL1 may be a new treatment target in chronic kidney disease.

## 4. Material and Methods

### 4.1. Animals

All animal experiments conducted in this study were approved by the University of Toronto Faculty of Medicine Animal Care Committee (protocol no. 20011495) per the Regulations of the Animals for Research Act in Ontario and the Guidelines of the Canadian Council on Animal Care. *Col4a3^-/-^* mice (stock no. 002908) on the 129X1/SvJ background, WT controls (stock no. 000691), and C57BL/6J mice (stock no.000664) were purchased from The Jackson Laboratory (Bar Harbor, ME, USA). Mice were housed at the Division of Comparative Medicine (University of Toronto, Toronto, ON, Canada) in a 12-h dark–light cycle and fed standard rodent diet (2018 Teklad global 18% protein) purchased from Envigo (Huntingdon, UK), with free access to water. Only male mice were used in this study. Mice were randomly assigned to control and treatment groups. Investigators were not blinded unless otherwise stated. Numbers of biological replicates are stated within figure legends.

### 4.2. Cell Culture

Immortalized human proximal tubule epithelial (HK-2) cells were cultured in Gibco DMEM/F-12 (Thermo Fisher Scientific, Waltham, MA, USA) supplemented with 10% fetal bovine serum (Thermo Fisher Scientific), 10 ng/mL epidermal growth factor (MilliporeSigma, Burlington, MA, USA), 5 μg/mL transferrin (MilliporeSigma), 5 μg/mL insulin (MilliporeSigma), 0.05 μM hydrocortisone (MilliporeSigma), 50 U/mL penicillin (Thermo Fisher Scientific), and 50 μg/mL streptomycin (Thermo Fisher Scientific). Cells were maintained at 37 °C with 5% CO2. HK-2 cells were subcultured in six-well plates and then starved of serum overnight. For phosphorylated ERK and p38 immunoblots, cells were treated with DMEM/F-12 medium for control and 125 ng/mL rhFSTL1 (Novus Biologicals NBP2-23056) in DMEM/F-12 medium for either 5, 10, 30, 60, or 120 min. For PARP and CASP3, cells were treated with DMEM/F-12 medium for control, 125 ng/mL rhFSTL1 in DMEM/F-12 medium for 16 h, or 1 μm of Naloxone in DMEM/F12 for 8 h the subsequently treated with 125 ng/mL rhFSTL1 in DMEM/F-12 medium for 16 h. For COX2, cells were treated with DMEM/F-12 medium for control or 125 ng/mL rhFSTL1 in DMEM/F-12 medium for 18 h. For COL1A1, cells were treated with DMEM/F-12 medium for control or 125 ng/mL rhFSTL1 in DMEM/F-12 medium for 48 h.

### 4.3. Immunoblotting

Total protein extract was transferred to a tube with 5× SDS sample loading buffer and boiled at 95 °C for 5 min. Proteins were separated by SDS-PAGE and then transferred onto PVDF membranes. Membranes were blocked and subsequently incubated with primary antibodies overnight at 4 °C. Primary antibodies were used at a dilution of 1:1000, except where otherwise indicated. The following rabbit primary antibodies were purchased from Cell Signaling Technology: phospho-p44/42 MAPK (ERK1/2; cat no. 9101), p44/42 MAPK (ERK1/2; cat no. 9102), phospho-p38 MAPK (1:500; cat no. 9211), p38 MAPK (1:500; cat no. 9212), COX2 (cat no. 12282), PARP (cat no. 9542), CASP3 (cat no. 9664). COL1a1 was purchased from Cedarlane (product code: CL50151AP-1) The mouse primary antibody for b-actin (1:4000; cat no. A5441) was purchased from MilliporeSigma. Membranes were incubated with HRP-conjugated goat anti-rabbit (cat no. 7074) and bands were detected by enhanced chemiluminescence with the Luminata Forte Western HRP Substrate (MilliporeSigma). Densitometry was performed with Scion Image (Scion Corporation, Frederick, MD). For FSTL1 membranes were blocked and subsequently incubated with FSTL1 (R&D system, cat no. AF1738) overnight at 4 °C at a dilution of 0.1 µg/mL. Membranes were then incubated with HRP-conjugated mouse anti-goat IgG antibody (merck-millipore). Bands were detected using (ImageQuant LAS 4000 mini, GE Healthcare) and Densitometry was performed with Scion Image (Scion Corporation, Frederick, MD, USA).

### 4.4. RNAscope^®^

RNA Chromogenic in situ hybridization Visualization of mRNA transcript was performed using RNAScope^®^ 2.5 (Advanced Cell Diagnostics, Hayward, CA, USA), according to the manufacturer’s instructions. A 20ZZ probe (RNAscope^®^ Target Probe C1) was designed and named Mm-Fstl1 targeting 100–1102 of NM_008047.5.

### 4.5. Luciferase

Cells were transfected with Renilla luciferase control reporter vector pRL-TK and a luciferase reporter for either NFκB or AP-1 vector and incubated with fresh growth medium (DMEM/F-12) for 24 h, and then starved of serum (serum-free DMEM/F-12) for 24 h. Cells were then treated with 125 ng/mL rhFSTL1 for 24 h. The control group was treated with serum free DMEM/F-12 for 24 h. Reporter activities were measured using the Promega, Madison Wisconsin dual-luciferase assay kit. The luciferase activity was normalized to the Renilla luciferase activity.

### 4.6. Quantitative PCR

Total RNA was purified using the RNeasy Mini Kit (Qiagen, Hilden, Germany) by following the manufacturer’s protocol. cDNA was synthesized from purified template RNA with the QuantiTect Reverse Transcription Kit (Qiagen). Quantitative PCR was performed with Applied Biosystems TaqMan Gene Expression Assays (Thermo Fisher Scientific) run on a ViiA 7 Real-Time PCR System (Thermo Fisher Scientific). The mouse TaqMan Gene Expression Assays that were used include: Mm00433371_m1; *Fstl1*, Mm00445273_m1; *Tlr4*, Mm01150153_m1; *Dip2a*, Mm00441242_m1; Ccl2, Mm00443258_m1; Tnfa, Mm00725412_s1; *Acta2*, Mm01178820_m1; *Tgfb1*, Mm00801666_g1; *Col1a1*, Mm01256744_m1; *Fn1*. Values were determined using the relative standard curve method. *Gapdh* served as the housekeeping gene.

### 4.7. Histological Staining

Three-micrometer formalin-fixed, paraffin-embedded kidney sections were used for periodic acid-Schiff (PAS), Masson’s trichrome (MTC), and α-SMA (SMA) staining. The rabbit primary antibody for α-SMA (cat no. ab5694) was purchased from Abcam. PAS and MTC as well as α-SMA were performed at the University Health Network Pathology Research Program Laboratory (Toronto, ON, Canada).

### 4.8. Heatmaps

Heatmaps were generated using the gplots package in RStudio version 3.5.2.

### 4.9. Unilateral Ureteral Obstruction (UUO)

Unilateral ureteral obstruction (UUO) 7 week old male C57BL/6J, mice were anesthetized with inhalational 3% isoflurane and administered analgesic (buprenorphine, 0.1 mg/kg s.c.). A midline dorsal incision was made to expose the left kidney ureter which was ligated with a 4–0 suture. The contralateral (right) kidney served as the control. Body temperature was maintained during the procedure using a 37 °C heating pad. Incisions were closed using 4–0 sutures. After 7 days, the mice were euthanized, and kidneys were harvested.

### 4.10. Data Collection and Study Cohort

Percutaneous kidney biopsies were obtained from patients after informed consent and with approval of the local ethics committees at each of the participating kidney centers. Written consent and assent were obtained. This covers all aspects of the study including clinical data, biospecimens and any derivatives. Clinical and gene expression information from patients is accessible in a non-identifiable manner. The University of Michigan institutional review board in the Department of Medicine (UMich IRBMED) is the institutional review board of record [55].

Biopsies from 211 subjects (78 females and 133 males) with nephrotic syndrome (FSGS, IgAN, MN) were microdissected into glomerular and tubulointerstitial components. Kidney biopsy tissue was manually micro-dissected to separate the tubulointerstitial compartment from the glomerular compartment. Total RNA was isolated, reverse transcribed, linearly amplified and hybridized on an Affymetrix 2.1 ST platform as described previously [55,56,57]. Gene expression was normalized, quantified, and annotated at the Entrez Gene level.

Visual assessment was performed according to the Nephrotic Syndrome Study Network Digital Pathology Scoring System (NDPSS), on de-identified whole slide images of kidney biopsies according to the NEPTUNE digital pathology protocol (NDPP) [55]. Visual quantitative assessment of IF and TA was reported as 0–100%. Pathological assessment of IF and TA was performed according.

Estimated glomerular filtration rate (eGFR) (mL/min/1.73 m^2^) was calculated using the Chronic Kidney Disease Epidemiology Collaboration (CKD-EPI) formula for participants ≥18 years old and the modified CKiD-Schwartz formula for participants <18 years old. Progression of eGFR was evaluated with a composite of 40% decline in eGFR from baseline or ESKD. ESKD was defined as the initiation of dialysis, receipt of kidney transplant or eGFR < 15 mL/min/1.73 m^2^ at two visits.

### 4.11. Statistics

Statistics Numbers of cohorts and *n* values for each experiment are indicated in figure legends. Unless stated otherwise, data are reported as the mean ± SEM. Statistical analyses were performed using GraphPad Prism 7 (GraphPad Software, San Diego, CA, USA). *p* values were determined by two-tailed Student’s *t*-tests for comparisons between two groups or one-way ANOVA with Tukey’s multiple comparisons tests for comparisons between three groups or more.

## Figures and Tables

**Table 1:** Clinical characteristic for Alport and wild-type mice.

	4 Weeks		7 Weeks	
	WT (*n* = 8)	KO (*n* = 8)	WT (*n* = 8)	KO (*n* = 8)
Body weight (g)	16.63 ± 0.74	17.60 ± 0.46	21.17 ± 0.50	19.51 ± 0.93
LKW(g)/BW (Kg)	7.08 ± 0.17	7.61 ± 0.33	7.43 ± 0.18	8.82 ± 0.17
RKW(g)/BW (Kg)	7.08 ± 0.17	7.69 ± 0.31	7.69 ± 0.07	8.84 ± 0.21
P_Cr_ (μMol/L)	16.80 ± 1.32	16.00 ± 1.00	18.25 ± 1.65	35.80 ± 14.06
UalbV (mg/dl)	20.45 ± 2.25	65.66 ± 6.33	20.14 ± 1.45	144.31 ± 12.84

Body weight, left kidney weight to body weight ratio, right kidney weight to body weight ratio, protein creatinine, and urinary albumin levels for 4 and 7 week old wild type (WT) and *Col4a3*^-/-^ (KO) mice (values are mean ± SEM).

**Table 2:** Demographic and clinical characteristics of patients with FSGS, IgAN, and MN.

	ALL	FSGS	IgAN	MN
Age	38.54 ± 1.364	32.62 ± 1.957	36.08 ± 2.664	50.89 ± 1.792
Sex (male/female)	(133/78)	(66/45)	(28/11)	(39/22)
BMI	28.51 ± 0.4938	27.39 ± 0.7284	28.24 ± 0.9308	30.72 ± 0.8484
Sitting Systolic	123.6 ± 1.209	122.7 ± 1.558	123.1 ± 2.67	125.5 ± 2.575
Sitting Diastolic	76 ± 0.8592	74.96 ± 1.212	75.85 ± 1.936	77.97 ± 1.557
Hematocrit %	39.08 ± 0.389	38.83 ± 0.5635	38.54 ± 0.8987	39.88 ± 0.654
eGFR	74.06 ± 2.214	72.93 ± 3.247	67.46 ± 5.559	80.32 ± 3.255
Centrally measured timed urine protein	243.8 ± 23.2	199.3 ± 27.74	119.9 ± 18.73	385.6 ± 52.7
Centrally measured timed urine creatinine	69.01 ± 3.536	69.07 ± 5.286	62.35 ± 5.869	72.86 ± 6.701
Centrally measured timed urine albumin	1778 ± 169	1492 ± 208.3	927 ± 146.7	2737 ± 382
Interstitial fibrosis (%)	18.71 ± 1.658	20.98 ± 2.496	20.65 ± 3.155	12.38 ± 2.179
Tubular atrophy (%)	17.48 ± 1.654	19.44 ± 2.489	19.62 ± 3.175	11.71 ± 2.22
Patient reached ESKD or 40% loss of eGFR (and eGFR<90)	50	27	10	13

FSGS, focal segmental glomerulosclerosis; IgAN, IgA nephropathy; MN, membranous glomerulonephropathy. BMI, body mass index; eGFR, estimated glomerular filtration rate. Values are mean ± SEM.

**Table 3:** Multiple linear regression analysis of *FSTL1* expression.

Model: y = *β*_0_ + *β*_1_*x*_1_ + *β*_2_*x*_2_ + *β*_3_*x*_3_ + *β*_4_*x*_4_ + *β*_5_*x_5_* + *β*_6_*x_6_*					
Predictor	Coefficient	Estimate	Standard Error	*t*-Statistic	*p*-Value
*FSTL1*	*β*_0_	4.0903	0.4391	9.3144	<0.001
Age	*β*_1_	−0.0099	0.0029	−3.4147	0.0008
Sex	*β*_2_	0.0976	0.1078	0.9054	0.3667
BPSS	*β*_3_	0.0035	0.0041	0.8616	0.3903
BPSD	*β*_4_	−0.0031	0.0057	−0.5522	0.5817
eGFR	*β*_5_	−0.0134	0.0018	−7.4402	<0.001
UPCR	*β*_6_	0.071	0.0131	5.4027	<0.001

Age; Sex; Systolic, sitting systolic blood pressure; Diastolic, sitting diastolic blood pressure; eGFR, estimated glomerular filtration rate; UPCR, centrally measured timed urinary protein creatinine ratio.

**Table 4:** *FSTL1* quartile expression. Baseline *FSTL1* mRNA levels divided into quartiles.

Quartile Analysis				
Quartile	1	2	3	4
Percentage (%)	18%	22%	18%	38%
*n*	9/49	11/49	9/49	18/48
Range	1.6–2.7	2.7–3.1	3.1–3.7	3.7–5.6

The number (*n*) and percentage of patients in each *FSTL1* quartile that reached the composite endpoint. The composite end point has two components: End Stage Kidney Failure (ESKD) or a 40 percent decrease in eGFR compared to baseline eGFR (with a baseline eGFR < 90 mls/min).
